# Current clinical applications and clinical evidence of Bojungikki-tang, a traditional East Asian multi-botanical preparation: a scoping review

**DOI:** 10.3389/fphar.2026.1831561

**Published:** 2026-07-10

**Authors:** Soo-Dam Kim, Boram Lee, Yang-Chun Park, Yee Ran Lyu

**Affiliations:** 1 KM Science Research Division, Korea Institute of Oriental Medicine, Daejeon, Republic of Korea; 2 Department of Internal Medicine, College of Korean Medicine, Daejeon University, Daejeon, Republic of Korea

**Keywords:** Bojungikki-tang, botanical drug, Buzhongyiqi decoction, clinical evidence, Hochuekkito

## Abstract

**Background:**

Bojungikki-tang (Buzhongyiqi decoction, Hochuekkito, BIT) has been used in East Asian medicine for conditions with fatigue, reduced appetite, and functional decline. In recent years, research on this preparation has expanded across diverse disease areas, yet its contemporary evidence landscape has not been systematically mapped. This scoping review aimed to characterize the scope, methodological features, outcome domains, botanical drug reporting, and safety reporting of studies published since 2015.

**Methods:**

A comprehensive search of PubMed, Embase, CENTRAL, and OASIS was conducted to identify studies evaluating BIT. Eligible studies included randomized controlled trials non-randomized trials, observational studies, and case-based reports published from 2015 onward. Data were charted on study design, disease system, intervention characteristics, botanical composition, formulation reporting, outcome domains, and adverse-event reporting, and synthesized descriptively.

**Results:**

Forty-seven studies involving 2,568 participants were included. RCTs accounted for 48.9%, while case-based studies comprised 27.7%. Oncology represented the largest disease cluster (27.7%), followed by urogenital (17.0%), neurologic (14.9%), and respiratory conditions (12.8%). BIT was used as monotherapy in 55.3% of studies and as an adjunct in 44.7%. Granule and decoction forms accounted for 89.4% of interventions. Outcomes assessed fatigue, appetite, quality of life, functional recovery, inflammatory and nutritional biomarkers, and treatment tolerance. Heterogeneity in formulation, comparators, outcomes, and safety reporting limited comparability.

**Conclusion:**

Recent studies suggest that BIT is investigated as a recovery-oriented intervention in conditions with immune–inflammatory stress and functional vulnerability. Greater standardization in botanical drug nomenclature, formulation definition, outcome frameworks, and harms reporting is needed for evidence-based botanical drug research.

## 1 Background

Bojungikki-tang (also known as Hochu-ekki-to or Buzhong Yiqi Decoction, BIT) is a classical East Asian multi-botanical preparation traditionally prescribed for states of reduced vitality characterized by fatigue, diminished appetite, and impaired recovery capacity. It is a multi-botanical preparation traditionally composed of *Astragali Radix*, *Ginseng Radix*, and other supporting botanical drugs, and is used to strengthen digestive function and promote systemic restoration during deficiency conditions (Hu et al., 2024). Its continued use across Korean Medicine, Chinese medicine, and Japanese Kampo medicine makes BIT a representative traditional East Asian multi-botanical preparation for examining how long-standing clinical use is being translated into contemporary human research. Because the preparation is commonly chosen to improve functional status rather than target a single disease entity, it has been applied across a wide range of clinical settings, including postoperative recovery, frailty-related syndromes, and supportive management of chronic illnesses (Hu et al., 2024; Kim et al., 2017; Guo et al., 2022). While this broad applicability has contributed to its sustained clinical relevance, it has also blurred the boundaries of its most evidence-supported indications, creating challenges for both clinical interpretation and research synthesis.

Despite widespread clinical use, routine decision-making surrounding BIT continues to depend largely on traditional indications and practitioner experience rather than clearly defined, evidence-based clinical contexts. Studies in older or frail populations have reported improvements in patient-centered outcomes such as quality of life and immune-related parameters, supporting a potential role in deficiency-related conditions (Satoh et al., 2005; Kuroiwa et al., 2004). More recent controlled studies have explored recovery, appetite, and activity outcomes in postoperative settings, again pointing toward functional benefits that matter to patients and caregivers (Watanabe and Maeda, 2022; Okugawa et al., 2024). Nevertheless, much of the existing evidence remains condition-specific, limited in scale, or confined to single healthcare systems, leaving clinicians without an integrated understanding of studied populations, BIT formulation variations, comparators, and outcome priorities.

An additional challenge arises from the fragmented and condition-specific nature of the existing clinical literature, much of which is limited by small sample sizes, heterogeneous designs, variable methodological quality, and a relative lack of rigorously conducted randomized controlled trials (RCTs), resulting in an insufficient body of high-quality integrated evidence. Evidence syntheses in selected disease areas demonstrate both therapeutic promise and methodological limitations. Meta-analytic findings in stable chronic obstructive pulmonary disease (COPD) indicate potential improvements in respiratory function, exercise tolerance, and quality of life, while consistently highlighting the need for more rigorous RCTs (Chen et al., 2016; Kwon et al., 2021). Similarly, pooled analyses in functional constipation suggest symptomatic benefit but are constrained by heterogeneity and variable study quality (Gong et al., 2018). Together, these findings reflect sustained clinical interest in BIT while underscoring the absence of a comprehensive, clinically oriented synthesis of human research evidence.

This scoping review aims to assemble an up-to-date, clinically oriented picture of how BIT has been studied in humans and where research activity is most concentrated. The goal is to summarize current clinical applications across diseases and age groups, identify which study designs dominate the landscape, and clarify how botanical drug composition, formulation types, comparators, safety reporting, and outcome measures vary in practice. This approach is particularly relevant because BIT is not a single standardized product, but a traditional multi-botanical preparation used in fixed commercial granules, decoctions, powders, pills, and modified prescriptions. By highlighting areas with repeated controlled testing alongside areas still supported mainly by exploratory clinical studies, the review will help clinicians interpret existing findings with appropriate caution and help researchers pinpoint feasible, high-impact questions for future controlled trials.

## 2 Methods

### 2.1 Study design and framework

This scoping review was designed based on the methodological framework originally proposed by Arksey and O’Malley (Arksey and O’Malley, 2005; Tricco et al., 2018), with additional guidance drawn from the Joanna Briggs Institute approach to strengthen methodological rigor (Peters et al., 2020). The reporting of the review followed the PRISMA Extension for Scoping Reviews (PRISMA-ScR) to ensure transparent and systematic presentation of the evidence identification and selection process. The protocol was prospectively registered on the Open Science Framework (OSF; https://osf.io/vphtm/overview; registered 23 September 2025). The review seeks to comprehensively map the clinical research landscape of BIT by summarizing study characteristics, populations, interventions, comparators, and outcome measures, thereby supporting future evidence synthesis and targeted clinical research planning. Consistent with scoping review methodology, formal risk-of-bias assessment was not conducted, as the purpose was to map the evidence rather than synthesize effect estimates.

### 2.2 Definition of preparations and taxonomic validation

BIT and its modified formulations evaluated in this review were defined as oral traditional East Asian multi-botanical preparations rather than a single standardized product. Botanical drug names were extracted as reported in the original studies, and full species names, author citations, family names, medicinal parts, and pharmacopeial drug names were standardized where identifiable in accordance with ConPhyMP recommendations and best-practice guidance for ethnopharmacological research (Heinrich et al., 2022; Rivera et al., 2014). Taxonomic validation was conducted using Plants of the World Online (Kew, https://powo.science.kew.org/) and Medicinal Plant Names Services (MPNS, https://mpns.kew.org). Because this study is a scoping review of published clinical studies, extraction methods, processing procedures, manufacturing conditions, batch information, certificates of analysis, and regulatory status were recorded only when reported in the original studies. The taxonomically validated botanical drug composition and other medicinal materials included in modified formulations are summarized in Supplementary Table S2.

### 2.3 Research questions

The review addresses the following questions: (1) To what extent has BIT been investigated in clinical research published since 2015, and across what populations (including disease categories, age groups, and geographic regions) and research designs (including case series, non-RCTs, and RCTs)? (2) What BIT formulations, comparators, and outcome measures have been used, and what recurring clinical clusters and intervention–outcome patterns can be identified to inform future evidence synthesis and clinical trial planning?

### 2.4 Eligibility criteria

Studies were included if they met the following criteria: (1) involved human participants without restrictions on age, disease category, or geographic location; (2) evaluated BIT as the primary intervention regardless of formulation type, preparation method, or modification; and (3) were published in peer-reviewed journals from January 2015 through December 2025. This time window was selected *a priori* to capture recent clinical use of BIT and contemporary standards in clinical trial design, intervention reporting, and safety reporting. All clinical study designs involving human subjects were considered eligible, including observational studies and controlled clinical trials. Studies were excluded if they met any of the following conditions: (1) non-human experimental research, including *in vitro* or animal studies; (2) study protocols, review articles, conference abstracts without full-text availability, or other non-original research; or (3) publications released prior to 2015.

### 2.5 Information sources and search strategy

A systematic search was conducted across PubMed, Embase, CENTRAL, and OASIS to capture both international biomedical literature and traditional medicine–focused publications relevant to BIT. The database search was performed on 16 December 2025. Search strategies incorporated a combination of controlled vocabulary terms and free-text keywords reflecting the formula and its commonly used variants. Representative search terms included “Bojungikki-tang,” “Bojungikgi-tang,” “Bu Zhong Yi Qi Tang,” “Buzhong Yiqi,” “Hochu-ekki-to,” “Hochuekkito,” and “TJ-41,” among others. These terms were adapted to the syntax of each database and combined using appropriate Boolean operators. No language restrictions were applied during the database search. Full-text articles were assessed when accessible, regardless of publication language. No additional manual reference screening was conducted beyond the predefined database search. The complete database-specific search strategies and full search strings are provided in Supplementary Table S1.

### 2.6 Study selection

All records retrieved from the database searches were imported into EndNote for reference management and duplicate removal. Two independent reviewers screened titles and abstracts according to the predefined eligibility criteria, followed by full-text assessment of potentially relevant studies. Discrepancies between reviewers were resolved through discussion, and when necessary, by consulting a third reviewer. The study selection process is presented using a PRISMA flow diagram, detailing the number of records identified, screened, included, and excluded, along with reasons for exclusion at each stage.

### 2.7 Data extraction and synthesis

Data extraction was independently performed by two reviewers using a standardized form developed *a priori* to ensure methodological consistency. Extracted information included author and publication year, country of study, study design, disease or condition investigated, mean or median participant age, formulation of BIT including dosage form and modifications, botanical drug composition, preparation or extraction information where available, commercial product information where available, concomitant medications or co-interventions, adverse-event reporting, interaction-related safety information, comparator type (if applicable), outcome measures, and key findings. Any disagreements during data extraction were resolved through discussion or, if required, by consultation with a third reviewer. For descriptive synthesis, BIT interventions were categorized by formulation type, modification status, and treatment role. Standardized BIT formulations were defined as fixed commercial products or fixed-dose preparations without patient-specific modification, whereas modified BIT formulations were defined as prescriptions in which botanical drugs were added, removed, or adjusted according to disease characteristics or patient presentation. BIT was also classified as monotherapy or adjunctive therapy according to whether it was used alone or in combination with conventional treatment, rehabilitation, acupuncture, pharmacopuncture, or other co-interventions. Outcome measures were grouped into patient-reported outcomes, functional measures, laboratory or biomarker outcomes, microbiologic or microbiome-related outcomes, treatment response outcomes, and safety outcomes. Frequency analyses were conducted to summarize the distribution of study designs, clinical indications, formulation types, comparator categories, and outcome domains. Narrative summaries complemented the descriptive findings to identify recurring research patterns and gaps within the clinical literature, thereby enabling systematic mapping of the evidence landscape.

## 3 Results

### 3.1 Study selection

**FIGURE 1:**
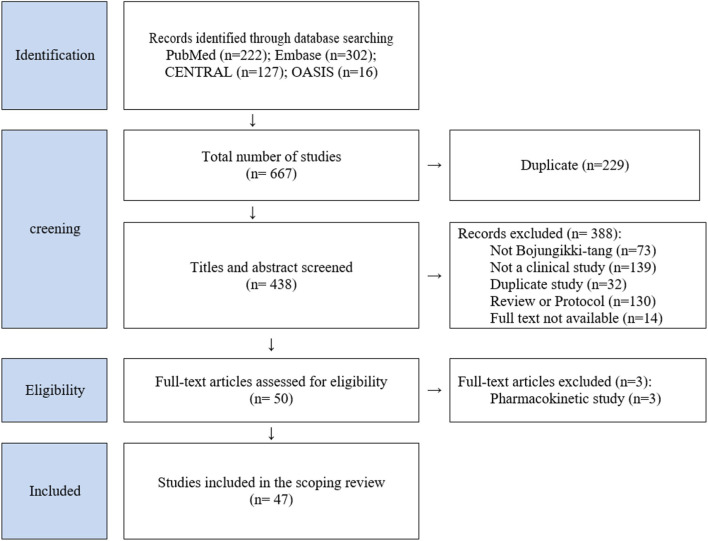
PRISMA study flow chart.

The database search yielded 667 records, including 222 from PubMed, 302 from Embase, 127 from CENTRAL, and 16 from OASIS. After removal of 229 duplicates, 438 records remained for title and abstract screening. Among these, 388 studies were excluded, including those not related to BIT (n = 73), non-clinical research (n = 139), duplicate publications (n = 32), review articles or study protocols (n = 130), and studies without available full text (n = 14). Fifty full-text articles were assessed for eligibility, of which three were excluded due to pharmacokinetic focus without clinical outcome evaluation. Ultimately, 47 clinical studies were included in the scoping review (Figure 1).

### 3.2 Characteristics of included studies

#### 3.2.1 Study design and geographic distribution

The 47 included studies involved a total of 2,568 participants. RCTs comprised 23 studies (48.9%) (Akita et al., 2019; Chen et al., 2024; Chen et al., 2018; Edahiro et al., 2022; Hamada et al., 2018; Hamada et al., 2022; Huang, 2015; Ko et al., 2025; Lee et al., 2025; Lee et al., 2024; Li et al., 2016; Lu et al., 2021; Okabe et al., 2019; Qian et al., 2024; Teng et al., 2016; Terai et al., 2020; Wang, 2016; Wang et al., 2021; Watanabe and Maeda, 2022; Wu et al., 2017; Xu et al., 2023; Yang, 2016; Zhang and Zhu, 2015), followed by case-based studies including case reports and case series (13 studies, 27.7%) (Kim et al., 2023; Kim and Keum, 2015; Lee et al., 2021; Lee et al., 2015; Lee et al., 2018; Lee, 2017; Lee et al., 2019; Li et al., 2021a; Minagawa et al., 2019; Park et al., 2025; Qiu et al., 2024; Seo et al., 2019; Takeuchi et al., 2017). Observational studies accounted for 9 studies (19.1%), consisting of 3 prospective and 6 retrospective designs (Hu et al., 2024; Huang et al., 2025; Kitahara et al., 2021; Kohno et al., 2021; Li et al., 2021b; Ni et al., 2021; Oh et al., 2023; Okugawa et al., 2024; Tokumasu et al., 2025). Only 2 studies (4.3%) employed non-RCT designs (Kim et al., 2024; Zhou et al., 2022). Geographically, China contributed 20 studies (42.6%), South Korea 13 studies (27.7%), Japan 13 studies (27.7%), and Taiwan 1 study (2.1%). RCTs were predominantly conducted in China and Japan, whereas case-based studies were more frequently reported in South Korea. Most studies were conducted in hospital-based settings, including 25 single-center hospital studies (53.2%) and 14 multicenter hospital studies (29.8%), while 7 studies (14.9%) were conducted in single-center clinic or community clinic settings and 1 study (2.1%) involved a mixed multicenter clinic/hospital setting. Funding sources were variably reported, with 21 studies (44.7%) reporting government or public funding, 2 studies (4.3%) reporting combined government/public and company funding, 4 studies (8.5%) reporting company funding, 2 studies (4.3%) reporting no funding, and 18 studies (38.3%) not reporting funding sources (Table 1).

**TABLE 1:** Characteristics of included clinical studies on Bojungikki-tang.

Study ID	Country	Study setting	Study design	Disease/Condition	Disease system	Sample size	Age (year)	Funding source
Akita et al. (2019)	Japan	Multicenter hospital	RCT	Chronic refractory wounds	Surgery	TG: 9/CG: 9	TG: 50/CG: 54	NR
Chen et al. (2024)	China	Single-center hospital	RCT	Postartum stress urinary incontinence (PSUI)	Urogenital	TG: 128/CG: 128	TG 27.83 ± 5.52/CG 27.81 ± 5.46	Government/public funding
Chen et al. (2018)	China	Single-center hospital	RCT	Early postpartum pelvic floor dysfunction (PFD)	Urogenital	TG: 93/CG: 93	TG 24.63 ± 2.05/CG 24.18 ± 2.12	NR
Edahiro et al. (2022)	Japan	Multicenter hospital	RCT	Myeloproliferative neoplasms (polycythemia vera, essential thrombocythemia, myelofibrosis)	Cancer	TG: 21/CG: 21	TG: 65 (IQR 50–71)/CG: 62 (IQR 56–65)	NR
Hamada et al. (2018)	Japan	Multicenter hospital	RCT	Chronic obstructive pulmonary disease (COPD)	Respiratory	TG: 18/CG: 15	TG: 75.3 ± 6.1/CG: 74.7 ± 7.1	Government/public + company
Hamada et al. (2022)	Japan	Multicenter hospital	RCT	COPD with apathy (mild depression excluded)	Respiratory	TG: 11/CG: 12	TG 72.5 ± 5.7/CG 76.4 ± 7.1	Government/public + company
Hu et al. (2024)	China	Single-center hospital	Retrospective observational study	Colorectal cancer after neoadjuvant chemotherapy and laparoscopic radical surgery with spleen-stomach qi deficiency	Cancer	TG: 50/CG: 50	TG 46.97 ± 14.21/CG 47.12 ± 13.37	NR
Huang (2015)	China	Single-center hospital	RCT	Type 2 diabetes mellitus	Metabolic	TG: 63/CG: 63	53.4 ± 3.2	NR
Huang et al. (2025)	China	Single-center hospital	Prospective observational study	Obstructive sleep apnea (OSA) with lung-spleen Qi deficiency	Respiratory	11	40.5 ± 10.2	Government/public funding
Kim et al. (2023)	South Korea	Single-center hospital	Case report	Postherpetic neuralgia	Neurologic	1	77	NR
Kim et al. (2024)	South Korea	Single-center hospital	Non-RCT	Long COVID (fatigue/Cognitive dysfunction)	Respiratory	BIT: 15/KOG: 15/CBD: 15	BIT: 43.80 ± 14.31/KOG: 45.33 ± 12.57/CBD: 51.33 ± 15.71	Government/public funding
Kim and Keum (2015)	South Korea	Single-center clinic	Case series	Chronic prostatitis/chronic pelvic pain syndrome (CP/CPPS)	Urogenital	11	48.9 ± 10.1 (34–63)	NR
Kitahara et al. (2021)	Japan	Single-center hospital	Retrospective observational study	MRSA colonization in acute stroke patients	Neurologic	TG: 41/CG: 32	BIT: 83.0 ± 9.2/Control: 82.2 ± 10.2	Company funding
Ko et al. (2025)	South Korea	Multicenter hospital	RCT	Advanced non-small-cell lung cancer (NSCLC) patients receiving atezolizumab	Cancer	TG: 14/CG: 14	TG: 73.36 ± 8.14/CG: 71.71 ± 7.98	Government/public funding
Kohno et al. (2021)	Japan	Multicenter hospital	Retrospective observational study	Vancomycin-resistant enterococci (VRE) colonization	Immune	TG: 16/CG: 106	TG: 69.9 ± 17.5/CG: 72.6 ± 12.2	Government/public funding
Lee et al. (2025)	South Korea	Single-center hospital	RCT	Anorexia in adults with atopic dermatitis	Immune	TG: 12/CG: 12	TG: 28.83 (95% CI: 25.43–32.24)/CG: 32.58 (95% CI: 23.37–41.80)	Government/public funding
Lee et al. (2021)	South Korea	Single-center clinic	Case report	Advanced NSCLC with brain & bone metastasis	Cancer	1	83	Government/public funding
Lee et al. (2015)	South Korea	Single-center hospital	Case report	Cervical cancer-related fatigue after concurrent chemoradiation therapy	Cancer	1	68	Government/public funding
Lee et al. (2018)	Taiwan	Single-center hospital	Case report	Multiple sclerosis with IFNβ-1a–induced flu-like symptoms	Neurologic	1	30	Government/public funding
Lee (2017)	South Korea	Single-center clinic	Case report	Chemotherapy-induced alopecia and general weakness in cholangiocarcinoma with liver metastasis	Cancer	1	70	NR
Lee et al. (2019)	South Korea	Single-center hospital	Case report	Sweating and palpitation after chemotherapy for primary central nervous system lymphoma	Cancer	1	41	Government/public funding
Lee et al. (2024)	South Korea	Single-center hospital	RCT	Persistent allergic rhinitis (PAR)	Immune	High-dose: 35/Standard-dose: 35/Placebo: 35	High: 36.74 ± 12.78/Std: 34.74 ± 11.93/Placebo: 33.03 ± 11.47	Government/public funding
Li et al. (2021a)	China	Multicenter hospital	Case report	Facioscapulohumeral muscular dystrophy (FSHD1)	Neurologic	1	15	NR
Li et al. (2021b)	China	Single-center hospital	Retrospective observational study	Juvenile ocular myasthenia gravis (JOMG)	Neurologic	BIT-only: 73/BIT + Pred: 33/BIT-instead: 29	BIT-only: 4.3 ± 3.6/BIT + Pred: 4.8 ± 3.5/BIT-instead: 5.8 ± 3.3	NR
Li et al. (2016)	China	Multicenter hospital	RCT	Postoperative NSCLC receiving radiochemotherapy	Cancer	TG: 40/CG: 40	TG 55.4 ± 9.6/CG 55.2 ± 9.8	Government/public funding
Lu et al. (2021)	China	Single-center hospital	RCT	Circumferential mixed hemorrhoids after PPH surgery	Surgery	TG: 60/CG: 60	TG: 42.8 ± 2.4/CG: 43.4 ± 2.7	NR
Minagawa et al. (2019)	Japan	Multicenter hospital	Case series	Cancer-related fatigue after starting enzalutamide in castration-resistant prostate cancer (CRPC)	Cancer	3	72, 69, 88 (mean: 76.3)	NR
Ni et al. (2021)	China	Single-center hospital	Prospective observational study	Obesity with polycystic ovary syndrome (PCOS)	Metabolic	15	27.6 (24–32)	Government/public funding
Oh et al. (2023)	South Korea	Single-center hospital	Retrospective observational study	Type 2 diabetes mellitus	Metabolic	15	81.53 ± 8.06	Government/public funding
Okabe et al. (2019)	Japan	Multicenter hospital	RCT	Stage II/III gastric cancer after curative resection	Cancer	TG: 55/CG: 55	TG: 65 (49–76)/CG: 64 (35–77)	Company funding
Okugawa et al. (2024)	Japan	Multicenter hospital	Retrospective observational study	Gastrointestinal cancer (majority metastatic)	Cancer	TG: 56	TG: 72.5 (66.5-81)	Company funding
Park et al. (2025)	South Korea	Single Korean medicine clinic	Case report	Adenomyosis	Urogenital	1	40	No funding
Qian et al. (2024)	China	Single-center hospital	RCT	Colorectal cancer	Cancer	BIT-placebo: 33/Placebo-BIT: 29	TG: 59.55 ± 8.98/CG: 61.79 ± 8.99	Government/public funding
Qiu et al. (2024)	China	Single-center hospital	Case report	Moderate generalized myasthenia gravis	Neurologic	1	50	Government/public funding
Seo et al. (2019)	South Korea	Single-center hospital	Case report	Urinary retention and dysuria after stroke	Neurologic	1	89	NR
Takeuchi et al. (2017)	Japan	Multicenter hospital	Case report	Late-onset hypogonadism syndrome	Urogenital	1	54	No funding
Teng et al. (2016)	China	Single-center clinic	RCT	Perimenopausal uterine bleeding	Urogenital	TG: 38/CG: 33	40–50	NR
Terai et al. (2020)	Japan	Multicenter clinic/hospital	RCT	Idiopathic male infertility	Urogenital	TG: 16/CG: 15	TG: 39.8 ± 5.8/CG: 39.4 ± 6.8	NR
Tokumasu et al. (2025)	Japan	Single-center hospital	Prospective observational study	Long COVID/Post-COVID-19 condition with general fatigue	Respiratory	20	42.9 ± 15.8	Government/public funding
Wang (2016)	China	Single-center community clinic	RCT	Postprandial fullness/dyspepsia-like symptoms	Gastrointestinal	TG: 35/CG: 35	NR	NR
Wang et al. (2021)	China	Multicenter hospital	RCT	Non–muscle-invasive bladder cancer after transurethral resection of bladder tumor (TURBT)	Cancer	TG: 35/CG: 35	TG: NR/CG: NR	NR
Watanabe and Maeda (2022)	Japan	Single-center hospital	RCT	Postoperative hip fracture (elderly)	Surgery	TG: 20/CG: 18	TG: 84.4 ± 8.9/CG: 85.1 ± 8.8	Company funding
Wu et al. (2017)	China	Single-center hospital	RCT	Postoperative anal fistula with food allergy	Immune	BIT: 6/Positive control: 6/Negative control: 6	22–65	Government/public funding
Xu et al. (2023)	China	Multicenter hospital	RCT	Hospital-acquired pneumonia with MDR bacteria	Respiratory	TG: 83/CG: 85	TG: 75.9 ± 12.3/CG: 76.5 ± 10.2	Government/public funding
Yang (2016)	China	Single-center community clinic	RCT	Geriatric reflux esophagitis	Gastrointestinal	TG: 64/CG: 58	TG: 67.1 (61-73)/CG: 65.2 (60-74)	NR
Zhang and Zhu (2015)	China	Single-center hospital	RCT	Cervical lesions post-, loop electrosurgical excision procedure (LEEP)	Surgery	TG: 30/CG: 30	TG: 38.29 ± 6.28/CG: 37.86 ± 7.86	Government/public funding
Zhou et al. (2022)	China	Single-center hospital	Non-RCT	Female stress urinary incontinence	Urogenital	90	50.0 ± 13.6	Government/public funding

BIT, Bojungikki-tang; CBD, Cheonwangbosim-dan; CG, control group; IQR, interquartile range; KOG, Kyungok-go; NR, not reported; RCT, randomized controlled trial; TG, treatment group.

#### 3.2.2 Disease system distribution and clinical focus

**FIGURE 2:**
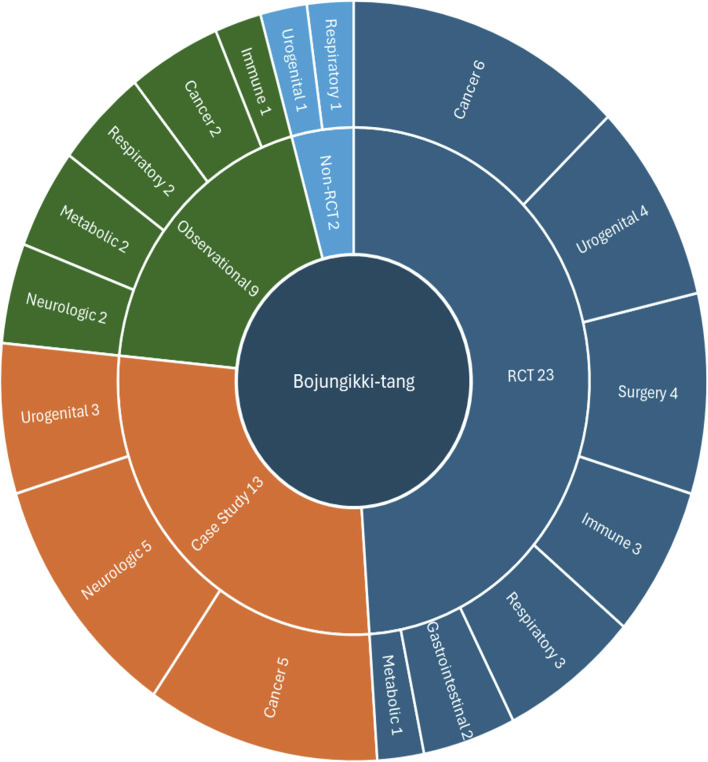
System-based mapping of clinical studies on Bojungikki-tang. RCT, randomized controlled trial.

For descriptive synthesis, indications were organized using a system-based clinical grouping approach according to the primary organ system or clinical domain involved. Cancer-related conditions represented the largest proportion of included studies, accounting for 13 studies (27.7%) (Edahiro et al., 2022; Hu et al., 2024; Ko et al., 2025; Lee et al., 2021; Lee et al., 2015; Lee, 2017; Lee et al., 2019; Li et al., 2016; Minagawa et al., 2019; Okabe et al., 2019; Okugawa et al., 2024; Qian et al., 2024; Wang et al., 2021). These investigations encompassed a broad spectrum of malignancies, including lung cancer, gastrointestinal cancers, cervical cancer, bladder cancer, and hematologic malignancies, reflecting a strong concentration of clinical research within oncology settings. Urogenital disorders comprised 8 studies (17.0%) (Chen et al., 2024; Chen et al., 2018; Kim and Keum, 2015; Park et al., 2025; Takeuchi et al., 2017; Teng et al., 2016; Terai et al., 2020; Zhou et al., 2022), followed by neurologic disorders with 7 studies (14.9%) (Kim et al., 2023; Kitahara et al., 2021; Lee et al., 2018; Li et al., 2021a; Li et al., 2021b; Qiu et al., 2024; Seo et al., 2019). Respiratory diseases were investigated in 6 studies (12.8%) (Hamada et al., 2018; Hamada et al., 2022; Huang et al., 2025; Kim et al., 2024; Tokumasu et al., 2025, Xu et al., 2023). Surgery-related conditions (Akita et al., 2019; Lu et al., 2021; Watanabe and Maeda, 2022; Zhang and Zhu, 2015) and immune-related conditions (Kohno et al., 2021; Lee et al., 2025; Lee et al., 2024; Wu et al., 2017) were each represented by 4 studies (8.5%). Metabolic disorders were examined in 3 studies (6.4%) (Huang, 2015; Ni et al., 2021; Oh et al., 2023), while gastrointestinal disorders were the least frequently studied category, comprising 2 studies (4.3%) (Wang, 2016; Yang, 2016) (Table 1; Figure 2).

### 3.3 Details in intervention characteristics

#### 3.3.1 Mode of administration and formulation

BIT was administered either as monotherapy or in combination with other therapeutic approaches. Monotherapy was employed in 26 studies (55.3%), whereas 21 studies (44.7%) evaluated BIT as an adjunct to conventional treatments, rehabilitation programs, pharmacological therapy, or other integrative interventions. Regarding formulation, granule preparations were most frequently used, appearing in 22 studies (46.8%) (Akita et al., 2019; Edahiro et al., 2022; Hamada et al., 2018; Hamada et al., 2022; Huang et al., 2025; Kim et al., 2024; Kitahara et al., 2021; Ko et al., 2025; Kohno et al., 2021; Lee et al., 2025; Lee et al., 2015; Lee, 2017; Lee et al., 2024; Minagawa et al., 2019; Oh et al., 2023; Okabe et al., 2019; Okugawa et al., 2024; Takeuchi et al., 2017; Terai et al., 2020; Tokumasu et al., 2025; Watanabe and Maeda, 2022; Wu et al., 2017), followed closely by decoction-based prescriptions in 20 studies (42.6%) (Chen et al., 2024; Chen et al., 2018; Hu et al., 2024; Huang, 2015; Kim et al., 2023; Lee et al., 2021; Li et al., 2021a; Li et al., 2021b; Li et al., 2016; Lu et al., 2021; Ni et al., 2021; Park et al., 2025; Qiu et al., 2024; Seo et al., 2019; Teng et al., 2016; Wang, 2016; Wang et al., 2021; Xu et al., 2023; Zhang and Zhu, 2015; Zhou et al., 2022). Powder and pill formulations were less common, each reported in 2 studies (4.3%) (Kim and Keum, 2015; Lee et al., 2018; Qian et al., 2024; Yang, 2016), while one study (2.1%) utilized a mixed formulation approach combining granules and decoctions (Lee et al., 2019) (Table 2).

**TABLE 2:** Characteristics of Bojungikki-tang interventions across included clinical studies.

Study ID	Intervention	Formulation	Composition	Dosage	Frequency	Treatment duration	Comparator
Akita et al. (2019)	Bojungikki-tang	Granules	Ginseng radix, atractylodis rhizoma, astragali radix, angelicae radix, zizyphi fructus, bupleuri radix, glycyrrhizae radix, zingiberis rhizoma, cimicifugae rhizoma, aurantii nobilis pericarpium	7.5 g/day	TID	12 weeks	Standard care only
Chen et al. (2024)	Bojungikki-tang + biofeedback electrical stimulation	Decoction	Astragali radix 18 g, angelicae radix 18 g, leonuri herba 18 g, codonopsis radix 16 g, dipsaci radix 15 g, atractylodis rhizoma 12 g, cimicifugae rhizoma 12 g, bupleuri radix 12 g, aurantii nobilis pericarpium 12 g, ligustici rhizoma 12 g, amomi fructus 10 g, chebulae fructus 10 g, corni fructus 10 g, glycyrrhizae radix praeparata 8 g	200 mL/day	BID	8 weeks	Pelvic floor muscle rehabilitation training only
Chen et al. (2018)	Modified bojungikki-tang + pelvic floor muscle exercise + biofeedback + electrical stimulation	Decoction	Codonopsis pilosula 16 g, atractylodes macrocephala 12 g, cimicifuga heracleifolia 12 g, astragalus membranaceus 18 g, angelica sinensis 18 g, citrus reticulata 12 g, glycyrrhiza uralensis (honey-fried) 8 g, leonurus japonicus 18 g, ligusticum chuanxiong 12 g, dipsacus asper 15 g, cornus officinalis 10 g, terminalia chebula 10 g, alpinia oxyphylla 10 g	150 mL/day	BID	6 weeks	Pelvic floor muscle exercise + biofeedback + electrical stimulation only
Edahiro et al. (2022)	Bojungikki-tang	Granules	Astragalus membranaceus, atractylodes lancea, panax ginseng, angelica acutiloba, bupleurum falcatum, zizyphus jujuba, citrus unshiu, glycyrrhiza uralensis, cimicifuga heracleifolia, zingiber officinale	7.5 g/day	TID	8 weeks	Standard care (no kampo)
Hamada et al. (2018)	Bojungikki-tang + pulmonary rehabilitation	Granules	Astragali radix (16.7%), atractylodis lanceae rhizome (16.7%), ginseng radix (16.7%), angelicae radix (12.5%), bupleuri radix (8.3%), zizyphi fructus (8.3%), aurantii nobilis pericarpium (8.3%), glycyrrhizae radix (6.3%), cimicifugae rhizome (4.2%), zingiberis rhizoma (2.0%)	7.5 g/day	TID	12 weeks	Pulmonary rehabilitation only
Hamada et al. (2022)	Bojungikki-tang + pulmonary rehabilitation	Granules	Astragali radix, atractylodis lanceae rhizoma, ginseng radix, angelicae radix, bupleuri radix, zizyphi fructus, aurantii nobilis pericarpium, glycyrrhizae radix, cimicifugae rhizoma, zingiberis rhizoma	7.5 g/day	TID	12 weeks	Pulmonary rehabilitation only
Hu et al. (2024)	Bojungikki-tang + conventional rehabilitation after laparoscopic surgery	Decoction	Angelica sinensis 15 g, dioscorea opposita 15 g, aurantii nobilis pericarpium 6 g, bupleurum chinense 6 g, glycyrrhiza uralensis 6 g, panax ginseng 9 g, cimicifuga heracleifolia 9 g, atractylodes macrocephala 9 g, amomi fructus 9 g, poria cocos 9 g, astragalus membranaceus 18 g	NR	BID	14 days	Conventional rehabilitation surgery only
Huang (2015)	Modified bojungikki-tang + metformin + acarbose	Decoction	Base formula: Angelica sinensis 12 g, atractylodes macrocephala 15 g, lycium barbarum 15 g, panax ginseng 10 g, rehmannia glutinosa 15 g, poria cocos 15 g, astragalus membranaceus 30 g, bupleurum chinense 12 g, cimicifuga heracleifolia 6 g, citrus reticulata 10 g.	NR	BID	8 weeks (2 cycles × 4 weeks)	Metformin + acarbose
Huang et al. (2025)	Bojungikki-tang	Granules	NR	NR	TID	3 months	Not applicable
Kim et al. (2023)	Modified bojungikki-tang + acupuncture + pharmacopuncture	Decoction	Base formula (per dose): Ginseng radix 12 g, astragali radix 12 g, atractylodis rhizoma alba 4 g, angelicae radix 4 g, aurantii nobilis pericarpium 4 g, glycyrrhizae radix 4 g, perillae herba 2 g, agastachis herba 2 g, zingiberis rhizoma 4 g, ziziphi fructus 4 g. modifications: Initially added ephedrae herba 2 g, aconiti lateralis radix 2 g, asiasari radix 1 g, later removed, astragali radix +4 g added	NR	TID	22 days (inpatient)	Not applicable
Kim et al. (2024)	Bojungikki-tang	Granules	Panax ginseng, atractylodes japonica, astragalus membranaceus, angelica gigas, zizyphus jujuba, bupleurum falcatum, citrus unshiu, glycyrrhiza uralensis, cimicifuga heracleifolia, zingiber officinale	7.5 g/day	BID	12 weeks + 24-week follow-up	Kyungok-go (KOG), cheonwangbosim-dan (CBD)
Kim and Keum (2015)	Modified bojungikki-tang	Powder	Astragalus membranaceus (astragali radix, 6 g), panax ginseng (ginseng radix, 4 g), glycyrrhiza uralensis (glycyrrhizae radix, 4 g), atractylodes macrocephala (atractylodis macrocephalae rhizoma, 4 g), fraxinus rhynchophylla (fraxini cortex, 2 g), angelica gigas (angelicae gigantis radix, 2 g), actaea/Cimicifuga spp. (cimicifugae rhizoma, 1.2 g), bupleurum falcatum (bupleuri radix, 1.2 g), plus massa medicata fermentata (amount NR), hordeum vulgare (hordei fructus germinatus, amount NR), crataegus pinnatifida (crataegi fructus, amount NR), proprietary “supp. A” (amount NR)	6 g/day	TID	Mean 5.7 ± 2.7 months (range 3–11 months)	Not applicable
Kitahara et al. (2021)	Bojungikki-tang	Granules	Astragalus membranaceus (JP astragalus root), atractylodes japonica (JP atractylodes rhizome), panax ginseng (JP ginseng), angelica acutiloba (JP Japanese angelica root), bupleurum falcatum (JP bupleurum root), zizyphus jujuba (JP jujube), citrus unshiu (JP citrus unshiu peel), glycyrrhiza uralensis (JP glycyrrhiza), cimicifuga simplex (JP cimicifuga rhizome), zingiber officinale (JP processed ginger)	7.5 g/day	TID	3 months	Standard care only
Ko et al. (2025)	Bojungikki-tang + atezolizumab	Granules	Ginseng radix 1.33 g, atractylodis rhizoma alba 1.33 g, astragali radix 1.33 g, angelicae gigantis radix 1.0 g, zizyphi fructus 0.67 g, bupleuri radix 0.67 g, citri unshius pericarpium 0.67 g, cimicifugae rhizoma 0.33 g, zingiberis rhizoma recens 0.17 g, glycyrrhizae radix et rhizoma 0.5 g (daily dose)	4 g/day	QD	9 weeks (3 cycles)	Placebo + atezolizumab
Kohno et al. (2021)	Bojungikki-tang	Granules	Astragalus membranaceus (JP astragalus root), atractylodes lancea (JP atractylodes rhizome), panax ginseng (JP ginseng), angelica acutiloba (JP Japanese angelica root), bupleurum falcatum (JP bupleurum root), zizyphus jujuba (JP jujube), citrus unshiu (JP citrus unshiu peel), glycyrrhiza uralensis (JP glycyrrhiza), cimicifuga simplex (JP cimicifuga rhizome), zingiber officinale (JP ginger)	7.5 g/day	TID	NR (administered until VRE-negative)	Probiotics ± standard care
Lee et al. (2025)	Bojungikki-tang	Granules	Panax ginseng 4.0 g, atractylodes japonica 4.0 g, astragalus membranaceus 4.0 g, angelica gigas 3.0 g, zizyphus jujuba 2.0 g, bupleurum falcatum 2.0 g, citrus unshiu 2.0 g, glycyrrhiza uralensis 1.5 g, cimicifuga heracleifolia 1.0 g, zingiber officinale 0.5 g (daily extract equivalent)	7.5 g/day	BID	8 weeks + 4-week follow-up	Waiting-list control (no active treatment)
Lee et al. (2021)	Gefitinib + modified bojungikki-tang	Decoction	Panax ginseng 24 g, astragalus membranaceus 24 g, liriope platyphylla 24 g, magnolia obovata 16 g, zingiber officinale 12 g, atractylodes japonica 8 g, angelica gigas 8 g, citrus reticulata 8 g, glycyrrhiza uralensis 8 g, pinellia ternata 8 g, ziziphus jujuba 8 g, cervus nippon 5 g, agastache rugosa 4 g, perilla frutescens 4 g	NR	TID	>78 months	Not applicable
Lee et al. (2015)	Bojungikki-tang	Granules	NR	7.5 g/day	TID	8 weeks	Not applicable
Lee et al. (2018)	Modified bojungikki-tang combined with salvia miltiorrhiza, ligusticum chuanxiong, angelica dahurica, and polygonum multiflorum thunb.	Powder	Astragali radix, atractylodis rhizoma, panacis ginseng radix, angelicae radix, bupleuri radix, zizyphi fructus, aurantii nobilis pericarpium, glycyrrhizae radix, cimicifugae rhizoma, zingiberis rhizoma, + salvia miltiorrhiza, ligusticum chuanxiong, angelica dahurica, polygonum multiflorum thunb.	9 g/day	TID	2 months	Not applicable
Lee (2017)	Bojungikki-tang + allergen-removed rhus verniciflua extract	Granules	Astragali radix 1.0 g, atractylodis rhizoma 1.0 g, ginseng radix 1.0 g, angelicae radix 0.75 g, bupleuri radix 0.5 g, zizyphi fructus 0.5 g, aurantii nobilis pericarpium 0.5 g, glycyrrhizae radix 0.375 g, cimicifugae rhizoma 0.25 g, zingiberis rhizoma 0.125 g	3.75g/day	TID	5 months	Not applicable
Lee et al. (2019)	Modified bojungikki-tang + Acupuncture	Decoction + Granules (hamsoapharm)	Ginseng radix 12 g, astragali radix 12 g, zingiberis rhizoma crudus 6 g, glycyrrhizae radix 4 g, atractylodis rhizoma alba 4 g, angelicae gigantis radix 4 g, citri pericarpium 4 g, jujubae fructus 4 g, amomi fructus 4 g, amomi rotundus fructus 4 g, alpiniae oxyphyllae fructus 4 g, agastachis herba 2 g, mori folium 2 g, bupleuri radix 0.14 g, cimicifugae rhizoma 0.08 g	NR	BID (decoction), TID (extract granule)	Decoction for 6 weeks), followed by extract granule for 2 weeks	Not applicable
Lee et al. (2024)	Bojungikki-tang	Granules	Atractylodis rhizoma alba 0.665 g, ginseng radix 0.665 g, astragali radix 0.665 g, angelicae gigantis radix 0.500 g, citri unshius pericarpium 0.335 g, bupleuri radix 0.335 g, zizyphi fructus 0.335 g, glycyrrhizae radix et rhizoma 0.250 g, cimicifugae rhizoma 0.165 g, zingiberis rhizoma 0.085 g	High-dose: 15 g/day; standard-dose: 7.5 g/day	TID	4 weeks	Placebo
Li et al. (2021a)	Modified bojungikki-tang	Decoction	Radix astragalus membranaceus 60 g, radix panax ginseng 10 g, rhizoma atractylodes macrocephala 30 g, radix angelica sinensis 10 g, pericarpium citrus reticulata 5 g, radix polygalae tenuifolia 5 g, rhizoma cimicifuga heracleifolia 5 g, radix bupleurum chinense 5 g, fructus lycium barbarum 10 g, semen cuscuta chinensis 15 g, fructus schisandra chinensis 5 g, poria cocos with hostwood 10 g, rhizoma zingiber officinale 10 g, fructus ziziphus jujube 10 g, radix glycyrrhiza uralensis 5 g	NR	QD	20 days (continued 6 months post-discharge)	Not applicable
Li et al. (2021b)	Bojungikki-tang	Decoction	NR	NR	NR	Mean: 1.3 years	Not applicable
Li et al. (2016)	Modified bojungikki-tang added to standard radio-/chemotherapy	Decoction	Astragalus membranaceus 60 g, hordeum vulgare germinated 25 g, *Triticum aestivum* germinated 25 g, atractylodes macrocephala 15 g, panax ginseng 15 g, citrus reticulata 10 g, bupleurum chinense 10 g, ligustrum lucidum 10 g, glycyrrhiza uralensis honey-fried 10 g, cimicifuga heracleifolia 10 g, eclipta prostrata 10 g, angelica sinensis 10 g, amomum villosum 6 g (+headache: Ligusticum chuanxiong & vitex trifolia 10 g, abdominal pain: Paeonia lactiflora 10 g, cough: Ophiopogon japonicus & schisandra chinensis 10 g)	NR	BID	From start of chemotherapy to 2 weeks post-chemotherapy (≈6 months total treatment course)	Standard radio-/chemotherapy only
Lu et al. (2021)	Modified bojungikki-tang + kangtai ointment (external)	Decoction	Paeonia lactiflora root 10 g, atractylodes macrocephala rhizome 10 g, angelica sinensis root 10 g, panax ginseng root 10 g, Bupleurum spp. root 9 g, actaea (cimicifuga) spp. rhizome 9 g, citrus reticulata pericarpium 6 g, glycyrrhiza uralensis (honey-fried) 5 g, astragalus membranaceus root 20 g	NR	BID	14 days	Topical chitosan gel (once daily)
Minagawa et al. (2019)	Bojungikki-tang	Granules	Astragalus membranaceus, atractylodes japonica, panax ginseng, angelica acutiloba, bupleurum falcatum, zizyphus jujuba, citrus unshiu, glycyrrhiza uralensis, cimicifuga japonica, zingiber officinale	7.5 g/day	TID	NR (follow-up until change in fatigue)	Not applicable
Ni et al. (2021)	Bojungikki-tang	Decoction	Astragalus membranaceus 30 g, poria cocos 15 g, codonopsis pilosula 15 g, atractylodes macrocephala 12 g, cimicifuga heracleifolia 9 g, bupleurum chinense 6 g, citrus reticulata 9 g, angelica sinensis 15 g, acorus tatarinowii 15 g, salvia miltiorrhiza 15 g, epimedium brevicornum 18 g, rehmannia glutinosa (prepared) 18 g	NR	BID	3 months	Not applicable
Oh et al. (2023)	Bojungikki-tang + glucose-lowering drugs	Granules	Bigwa BIT: Astragalus membranaceus 2.84 g, atractylodes macrocephala 1.90 g, gardenia jasminoides 1.90 g, angelica gigas 1.90 g, glycyrrhiza uralensis 1.90 g, citrus reticulata 1.90 g, liriope platyphylla 1.90 g, magnolia biondii 1.90 g, panax ginseng 1.90 g, cimicifuga heracleifolia 0.95 g, asarum sieboldii 0.95 g, bupleurum falcatum 0.95 g Hanshin BIT: Atractylodes macrocephala 1.25 g, panax ginseng 1.25 g, glycyrrhiza uralensis 1.25 g, astragalus membranaceus 1.88 g, angelica gigas 0.63 g, citrus reticulata 0.63 g, cimicifuga heracleifolia 0.63 g, bupleurum falcatum 0.38 g Kracie BIT: Atractylodes macrocephala 2.0 g, panax ginseng 2.0 g, glycyrrhiza uralensis 0.75 g, astragalus membranaceus 2.0 g, angelica gigas 1.5 g, citrus reticulata 1.0 g, cimicifuga heracleifolia 0.5 g, bupleurum falcatum 1.0 g, zingiber officinale 0.25 g, ziziphus jujuba 1.0 g	7.5-10 g/day	BID or TID	1–324 days (mean: 30.9 days)	Not applicable
Okabe et al. (2019)	Bojungikki-tang + S-1 adjuvant chemotherapy	Granules	10-Herb formula: Astragalus membranaceus, atractylodes macrocephala, panax ginseng, angelica acutiloba, bupleurum chinense, ziziphus jujuba, citrus unshiu, glycyrrhiza uralensis, cimicifuga heracleifolia, zingiber officinale (dosage not reported)	7.5 g/day	QD	1 year	S-1 alone
Okugawa et al. (2024)	Bojungikki-tang	Granules	Astragalus membranaceus 4.0 g, atractylodes lancea 4.0 g, panax ginseng 4.0 g, angelica acutiloba 3.0 g, bupleurum chinense 2.0 g, ziziphus jujuba 2.0 g, citrus unshiu 2.0 g, glycyrrhiza uralensis 1.5 g, cimicifuga heracleifolia 1.0 g, zingiber officinale 0.5 g	7.5 g/day	TID	≥3 months	Not applicable
Park et al. (2025)	Modified bojungikki-tang	Decoction	Astragalus membranaceus 4 g, angelica gigas 4 g, Cervus elaphus (cervi cornus colla) 1.67 g, panax ginseng 1.33 g, atractylodes macrocephala 1.33 g, glycyrrhiza uralensis 1.33 g, Equus asinus (asini corii colla) 1.33 g, rehmannia glutinosa 1.33 g, cimicifuga heracleifolia 0.67 g, bupleurum falcatum 0.67 g, citrus unshiu 0.67 g, poria cocos 0.67 g, alisma orientale 0.67 g, zingiber officinale 0.27 g	NR	TID (first 24 weeks), then BID	12 months +12-month follow-up	Not applicable
Qian et al. (2024)	Bojungikki-tang	Pills	Astragalus membranaceus, codonopsis pilosula, atractylodes macrocephala, angelica sinensis, cimicifuga foetida, bupleurum chinense, citrus reticulata (pericarpium citri reticulatae), glycyrrhiza uralensis	30 pills/day	TID	6 chemotherapy cycles with 7-day washout after 3 cycles	Placebo
Qiu et al. (2024)	Modified bojungikki-tang	Decoction	Astragalus membranaceus 80 g, angelica sinensis (stir-fried) 12 g, atractylodes macrocephala (stir-fried) 30 g, codonopsis pilosula 30 g, citrus reticulata 6 g, glycyrrhiza uralensis 9 g, panax ginseng 15 g, cimicifuga foetida 9 g, bupleurum chinense 6 g, dioscorea opposita (stir-fried) 6 g, epimedium brevicornum 30 g, coix lacryma-jobi (stir-fried) 30 g, lablab purpureus (stir-fried) 15 g, euryale ferox (stir-fried) 15 g, bombyx batryticatus 9 g, scolopendra subspinipes 2 g	NR	Initially BID → then QD	30 months	Not applicable
Seo et al. (2019)	Bojungikki-tang	Decoction	Astragali radix 12 g, ginseng radix 12 g, atractylodis rhizoma alba 12 g, glycyrrhizae radix 12 g, angelicae gigantis radix 8 g, aurantii nobilis pericarpium 4 g, cimicifugae rhizoma 4 g, bupleuri radix 4 g	150 mL/day	TID	10 days	Not applicable
Takeuchi et al. (2017)	Bojungikki-tang	Granules	NR	7.5 g/day	NR	NR	Not applicable
Teng et al. (2016)	Modified bojungikki-tang + norethindrone	Decoction	Astragalus membranaceus 40 g, atractylodes macrocephala (bran-fried) 15 g, citrus reticulata 10 g, cimicifuga foetida 15 g, bupleurum chinense 10 g, codonopsis pilosula 20 g, glycyrrhiza uralensis (honey-fried) 10 g, pueraria lobata 20 g, dipsacus asper 15 g	NR	BID	3 months	Norethindrone tablets
Terai et al. (2020)	Bojungikki-tang	Granules	NR	7.5 g/day	TID	12 weeks	Antioxidant supplement
Tokumasu et al. (2025)	Bojungikki-tang	Granules	JUNKOU formulation: Ginseng 4.0 g, jujube 2.0 g, atractylodis rhizoma 4.0 g, bupleuri radix 2.0 g, astragali radix 4.0 g, glycyrrhizae radix 1.5 g, angelicae gigantis radix 3.0 g, zingiberis rhizoma 0.5 g, aurantii nobilis pericarpium 2.0 g, cimicifugae rhizoma 1.0 g. TSUMURA formulation: Astragali radix 4.0 g, atractylodis lanceae rhizoma 4.0 g, ginseng radix 4.0 g, angelicae gigantis radix 3.0 g, bupleuri radix 2.0 g, jujubae fructus 2.0 g, aurantii nobilis pericarpium 2.0 g, glycyrrhizae radix 1.5 g, cimicifugae rhizoma 1.0 g	7.5 g/day	BID or TID	8 weeks	Not applicable
Wang (2016)	Modified bojungikki-tang	Decoction	Codonopsis pilosula 24 g; astragalus membranaceus 24 g; atractylodes macrocephala (fried) 24 g; angelica sinensis 12 g; citrus reticulata pericarp 12 g; bupleurum chinense 6 g; Cimicifuga spp. 6 g; glycyrrhiza uralensis (honey-fried) 9 g	400 mL	BID	1 month	Domperidone (oral), TID, 1 month
Wang et al. (2021)	Bojungikki-tang + THP bladder perfusion	Decoction	Astragalus membranaceus (root) 25 g, panax ginseng (root) 10 g, angelica gigas (root) 10 g, glycyrrhiza uralensis (root) 6 g, atractylodes macrocephala (rhizome) 10 g, cimicifuga foetida (rhizome) 10 g, citrus reticulata (pericarp) 10 g, bupleurum chinense (root) 10 g	NR	BID	12 months	THP (pirarubicin) bladder perfusion alone
Watanabe and Maeda (2022)	Bojungikki-tang	Granules	Astragalus membranaceus 2.5 g, atractylodes lancea 2.5 g, panax ginseng 2.5 g, angelica acutiloba 1.88 g, bupleurum falcatum 1.25 g, zizyphus jujuba 1.25 g, citrus unshiu peel 1.25 g, glycyrrhiza uralensis 0.94 g, cimicifuga simplex 0.63 g, zingiber officinale 0.30 g (per 7.5 g extract)	7.5 g/day	TID	From postoperative day 3 until discharge (∼6–10 weeks)	Usual postoperative rehabilitation only (no BIT)
Wu et al. (2017)	Bojungikki-tang	Granules	Astragalus membranaceus 15 g, codonopsis pilosula 15 g, atractylodes macrocephala 10 g, angelica sinensis 6 g, citrus tangerina peel 6 g, bupleurum chinense 5 g, cimicifuga foetida 5 g, glycyrrhiza uralensis (prepared) 5 g	200 mL/day	BID	2 weeks	Positive control: No BIT (food allergy only); Negative control: No food allergy, no BIT
Xu et al. (2023)	Bojungikki-tang + conventional western medicine	Decoction	Astragali radix, ginseng radix, glycyrrhizae radix, atractylodis macrocephalae rhizoma, angelicae sinensis radix, citri reticulatae pericarpium, cimicifugae rhizoma, bupleuri radix	200 mL/day	QD	28 days	Conventional western medicine
Yang (2016)	Mosapride + bojungikki-tang	Pills	NR	9 g/day	TID	30 days	Mosapride alone
Zhang and Zhu (2015)	Modified bojungikki-tang + topical powder (Xueping capsule content)	Decoction	Astragalus membranaceus 50 g, codonopsis pilosula 20 g, atractylodes macrocephala 15 g, glycyrrhiza uralensis 6 g, angelica sinensis 8 g, citrus reticulata 12 g, poria cocos 15 g, bupleurum chinense 6 g, cimicifuga foetida 6 g, sanguisorba officinalis 12 g, rubia cordifolia 12 g	400 mL/day	BID	7 days	Metronidazole topical powder only
Zhou et al. (2022)	Modified bojungikki-tang	Decoction	Astragalus membranaceus 15 g, atractylodes macrocephala (fried) 15 g, citrus reticulata 6 g, cimicifuga foetida 6 g, bupleurum chinense 9 g, ginseng radix 15 g, glycyrrhiza uralensis 3 g, angelica sinensis 12 g, alpinia oxyphylla 15 g, mantidis ootheca 9 g, eucommia ulmoides 15 g, lindera aggregata 6 g, rosa laevigata michx 15 g (modifications allowed)	NR	BID	4 weeks	Not applicable

BID, twice daily; BIT, Bojungikki-tang; NR, not reported; QD, once daily; TID, three times daily.

#### 3.3.2 Treatment duration and exposure patterns

Treatment duration varied substantially across studies, ranging from short-term courses of 1 week to extended interventions lasting up to 30 months. Most clinical trials implemented treatment periods between 4 and 12 weeks, reflecting a common timeframe for evaluating functional and symptomatic outcomes. Several studies reported long treatment or observation periods of 6 months or more, but explicit follow-up beyond the active treatment period was reported in only 3 studies (6.4%), limiting assessment of whether functional or symptomatic improvements were sustained after discontinuation of BIT (Table 2).

#### 3.3.3 Composition of Bojungikki-tang and modified formulations

Across the included studies, a consistent core composition corresponding to the classical ten-botanical drug BIT preparation was repeatedly employed, particularly in standardized granule-based preparations. This canonical structure, typically consisting of *Astragali Radix*, *Ginseng Radix*, *Atractylodis Rhizoma*, *Angelicae Radix*, *Bupleuri Radix*, *Zizyphi Fructus*, *Aurantii Nobilis Pericarpium*, *Glycyrrhizae Radix*, *Cimicifugae Rhizoma*, and *Zingiberis Rhizoma*, was used with minimal or no variation in multiple studies, largely those using commercial formulations in South Korea and Japan (Akita et al., 2019; Edahiro et al., 2022; Hamada et al., 2018; Hamada et al., 2022; Kitahara et al., 2021; Kohno et al., 2021; Minagawa et al., 2019; Okabe et al., 2019; Okugawa et al., 2024; Watanabe and Maeda, 2022; Kim et al., 2024; Ko et al., 2025; Lee et al., 2025; Lee, 2017; Tokumasu et al., 2025). Beyond this standardized formulation, the majority of studies implemented modified versions that preserved the core botanical drugs while incorporating additional botanical drugs to address disease-specific pathophysiology. Frequently added botanical drugs included *Codonopsis Radix* and *Leonuri Herba* (Chen et al., 2024; Chen et al., 2018; Ni et al., 2021; Wang, 2016; Wu et al., 2017; Zhang and Zhu, 2015), *Salviae Radix* and *Ligustici Rhizoma* (Chen et al., 2024; Chen et al., 2018; Lee et al., 2018; Li et al., 2016; Ni et al., 2021), *Dipsaci Radix* and other gynecologic-supporting botanical drugs (Chen et al., 2024; Chen et al., 2018; Teng et al., 2016), *Rehmanniae Radix* and *Poria cocos* (Hu et al., 2024; Huang, 2015; Ni et al., 2021; Park et al., 2025; Zhang and Zhu, 2015), *Epimedii Herba* and *Dioscoreae Rhizoma* (Hu et al., 2024; Ni et al., 2021; Qiu et al., 2024), and various digestive-supporting botanical drugs such as *Amomi Fructus* and related adjuncts (Chen et al., 2024; Hu et al., 2024; Kim and Keum, 2015; Lee et al., 2019; Li et al., 2016). The extent of modification varied considerably, ranging from minor additions to complex prescriptions containing more than fifteen medicinal materials (Li et al., 2021a; Ni et al., 2021; Qiu et al., 2024). Several studies explicitly allowed flexible adjustment of botanical drugs based on patient presentation, reflecting individualized traditional prescribing practices (Kim et al., 2023; Li et al., 2016; Zhou et al., 2022). In contrast, a subset of studies did not report detailed compositional information (Huang et al., 2025; Lee et al., 2015; Li et al., 2021b; Takeuchi et al., 2017; Terai et al., 2020; Yang, 2016), limiting precise assessment of formulation structure and dosage characteristics in those cases (Table 2).

### 3.4 Outcome domains and reporting patterns

Across the included studies, outcome selection was diverse and reflected the wide range of clinical contexts in which BIT was evaluated. Symptom-focused patient-reported measures and condition-specific clinical scores were used most often, including pain and neuropathic symptom instruments (Kim et al., 2023; Kim and Keum, 2015), pelvic and urinary symptom measures for postpartum or female urinary conditions (Chen et al., 2024; Chen et al., 2018; Zhou et al., 2022), and urinary retention–related monitoring in post-stroke care (Seo et al., 2019). Several studies also applied syndrome- or symptom-based scoring approaches alongside functional indices, particularly in integrative medicine settings (Hu et al., 2024; Li et al., 2016). Symptom trajectory and supportive care outcomes were also reported, including fatigue-related measures (Minagawa et al., 2019), general weakness and hair regrowth with laboratory monitoring (Lee, 2017), and autonomic or sleep-related symptom scoring with appetite/weight tracking (Lee et al., 2019).

Quality-of-life (QOL) outcomes were commonly assessed using generic and disease-specific tools, such as EORTC QLQ-C30, SF-36, EQ-5D/EQ-VAS, dermatology-related measures, and rhinitis-specific QOL instruments (Edahiro et al., 2022; Hamada et al., 2018; Hu et al., 2024; Kim et al., 2023; Kim et al., 2024; Lee et al., 2025; Lee et al., 2015; Lee et al., 2018; Lee et al., 2024; Li et al., 2016; Park et al., 2025; Qian et al., 2024; Watanabe and Maeda, 2022). Functional performance and activity endpoints were also frequently reported, particularly in respiratory and postoperative populations, including 6-min walking distance, step counts, dyspnea indices, physical activity metrics, and rehabilitation-related milestones (Hamada et al., 2018; Hamada et al., 2022; Watanabe and Maeda, 2022). Disease-specific functional scales were additionally used in neuromuscular and neurologic conditions, including strength-based assessments and validated disease scores (Kitahara et al., 2021; Li et al., 2021a; Li et al., 2021b; Qiu et al., 2024).

Laboratory and biomarker outcomes were incorporated across indications and included inflammatory markers (e.g., CRP and cytokines), nutritional indices (e.g., albumin and related measures), immune-related profiles (cell subsets and immunoglobulins), and metabolic or endocrine parameters (Akita et al., 2019; Hu et al., 2024; Huang, 2015; Ko et al., 2025; Lee et al., 2025; Li et al., 2016; Oh et al., 2023; Okugawa et al., 2024; Takeuchi et al., 2017; Terai et al., 2020). Infection-related studies also used microbiologic and clinical endpoints such as colonization status, febrile infectious events, and pneumonia severity or pathogen eradication indices (Kitahara et al., 2021; Kohno et al., 2021, Xu et al., 2023). Microbiome-related endpoints were reported in several studies, including gut microbiota composition and fecal metabolomic profiling, as well as wound-flora characterization in postoperative settings (Huang et al., 2025; Lee et al., 2025; Ni et al., 2021; Wu et al., 2017). In oncology-focused studies, outcomes extended beyond symptom and QOL measures to include performance status, tumor markers, and treatment response or survival-related endpoints (Hu et al., 2024; Ko et al., 2025; Lee et al., 2021; Okabe et al., 2019; Okugawa et al., 2024; Qian et al., 2024; Wang et al., 2021). Postoperative and wound-healing studies more often prioritized recovery-oriented endpoints such as time to wound healing, duration of discharge or bleeding, and pain resolution (Akita et al., 2019; Lu et al., 2021; Wu et al., 2017; Zhang and Zhu, 2015). Several gastrointestinal and gynecologic studies reported pragmatic effectiveness outcomes, including total effective rate and recurrence during follow-up (Teng et al., 2016; Wang, 2016; Yang, 2016). Overall, outcomes clustered around symptom burden, functional recovery, and QOL, but the breadth of instruments and reporting approaches limited direct comparability across studies, even within the same disease system (Table 3).

**TABLE 3:** Outcome measures, main findings, and safety reporting of included studies.

Study ID	Outcome measures	Main findings	Safety (AE)
Akita et al. (2019)	1. DESIGN-R score; 2. Albumin; 3. CRP; 4. IL-6	1.100% (8/8) vs. 33.3%; 2.4.0 g/dL vs. 3.6 g/dL; 3.0.7 mg/dL vs. 5.0 mg/dL; 4.6.8 pg/mL vs. 12.2 pg/mL	NR
Chen et al. (2024)	1. ICIQ-SF score; 2. Pelvic floor muscle strength; 3. Vaginal systolic pressure (VSP), resting pressure (VRP), duration; 4. Urodynamic parameters (ALPP, MUCP, Qmax, BC); 5. Total effective rate; 6. I-QOL score	1. ICIQ-SF: TG significantly lower vs. CG (p < 0.05); 2. Pelvic floor muscle strength: TG significantly higher vs. CG (p < 0.05); 3. VSP/VRP/duration: all significantly improved in TG vs. CG (p < 0.05); 4. Urodynamics (ALPP, MUCP, Qmax, BC): TG significantly improved vs. CG (p < 0.05); 5. Total effective rate: TG 125/128 (97.66%) vs. CG 100/128 (78.12%) (p < 0.001); 6. I-QOL: TG significantly higher vs. CG (p < 0.05)	NR
Chen et al. (2018)	1. Pelvic floor muscle strength: EMG (μV), duration (s), vaginal pressure (cmH_2_O), EMG score; 2. Urinary incontinence improvement rate; 3. Sexual life quality improvement rate; 4. TGF-β1; 5. MMP-2; 6. TIMP-2	1. Pelvic floor muscle strength outcomes were significantly improved in TG (all p < 0.05); 2. Urinary incontinence improvement rate was higher in TG (p < 0.05); 3. Sexual life quality improvement rate was higher in TG (p < 0.05); 4. TGF-β1 was significantly higher in TG (p < 0.05); 5. MMP-2 was significantly lower in TG (p < 0.05); 6. TIMP-2 was significantly higher in TG (p < 0.05)	NR
Edahiro et al. (2022)	1. MPN-SAF TSS; 2. ≥50% improvement in MPN-SAF TSS; 3. ≥20% improvement in MPN-SAF TSS; 4. EORTC QLQ-C30 (global health status/QOL, MID ≥10-point improvement)	1. MPN-SAF TSS: TG 23→17→18 vs. CG 22→∼22→∼22 (p = 0.299; no significant difference); 2. ≥50% improvement: TG 19% (4w), 20% (8w) vs. CG 16% (4w), 21% (8w); 3. ≥20% improvement: TG 57% (4w), 55% (8w) vs. CG 26% (4w), 26% (8w); 4. QOL MID ≥10-point improvement: TG 52.9% (4w) vs. CG 33.3% (4w). TG 26.7% (8w) vs. CG 40% (8w)	Grade 1 liver dysfunction (1/21, 4.8%), resolved after stopping
Hamada et al. (2018)	1. 6MWD; 2. Body weight; 3. %IBW; 4. mMRC dyspnea; 5. VAS-dyspnea; 6. VAS-fatigue; 7. CAT total	1. No significant between-group difference was observed in 6MWD; 2. Body weight increased in TG (p < 0.05); 3. %IBW increased in TG (p < 0.05); 4. mMRC dyspnea improved in TG (p < 0.05); 5. VAS-dyspnea improved in TG (p < 0.05); 6. VAS-fatigue improved in TG (p < 0.05); 7. CAT total improved in TG (p < 0.005)	No AE related to TJ-41; 1 death (abdominal aneurysm, unrelated); 1 pneumonia (CG); 2 COPD exacerbations (CG)
Hamada et al. (2022)	1. Apathy scale (AS); 2. Total number of steps; 3. PHQ-9; 4. VAS (dyspnea); 5. CAT (energy)	1. Apathy scale improved in TG compared with CG (p = 0.049); 2. Total number of steps was significantly higher in TG (p = 0.013); 3. PHQ-9 improved in TG (p = 0.031); 4. VAS-dyspnea improved in TG (p = 0.021); 5. CAT-energy improved in TG (p = 0.026)	1 mild liver dysfunction, 1 lumbar spinal canal stenosis; causal relation to TJ-41 unknown
Hu et al. (2024)	1. TCM syndrome scores (abdominal bloating, fatigue, emaciation, loose stool, loss of appetite, vomiting); 2. KPS score; 3. Immune function (CD3^+^, CD4^+^, CD8^+^, CD4^+^/CD8^+^, NK cells, IgG, IgA, IgM); 4. Inflammatory mediators (IL-10, IL-6, TNF-α, CRP); 5. Tumor markers (CEA, CA19-9, VEGF, TSGF); 6. SF-36 QOL	1. TCM syndrome scores were significantly improved compared with control (all p < 0.001); 2. KPS was significantly higher in the treatment group (p < 0.001); 3. Immune function indices were significantly improved (all p < 0.001); 4. Inflammatory mediators were significantly improved (all p < 0.001); 5. Tumor markers were significantly lower in the treatment group (all p < 0.001); 6. SF-36 QOL was significantly improved (p < 0.001)	TG: 6/50 (12.0%) – nausea (1), bloating (1), infection (2), fistula (1), gastric retention (1); CG: 15/50 (30.0%) – nausea (3), vomiting (3), abdominal pain (4), infection (3), fever (2)
Huang (2015)	1. FPG, 2hPG, HbA1c; 2. Glycemic control rate	1. FPG: 6.5 ± 1.5 vs. 7.5 ± 1.2 mmol/L 2hPG: 8.5 ± 2.2 vs. 10.2 ± 2.0 mmol/L. HbA1c: 6.9% ± 1.4% vs. 7.7% ± 1.3%; 2. Glycemic control rate: 93.7% (59/63) vs. 81.0% (51/63)	NR
Huang et al. (2025)	1. Apnea–Hypopnea index (AHI); 2. Micro-arousal index; 3. Minimum oxygen saturation (%); 4. Average oxygen saturation (%); 5. Gut microbiota abundance (alistipes, AR/PCNA pathway)	1. AHI: 61.2 ± 23.6 → 40.3 ± 19.2 (p = 0.0338); 2. Micro-arousal index: 56.6 ± 21.3 → 36.7 ± 21.1 (p = 0.0396); 3. Minimum SpO_2_: 66.3 ± 11.8 → 83.9 ± 4.4 (p = 0.0002); 4. Average SpO_2_: 88.9 ± 5.1 → 94.6 ± 2.0 (p = 0.0025); 5. Gut microbiota: ↑ *Alistipes*, activation of AR and PCNA gene pathways, improved flora balance (p < 0.05)	NR
Kim et al. (2023)	1. NRS; 2. SF-MPQ; 3. EQ-5D; 4. LANSS	1. NRS: 7 → 4; 2. SF-MPQ (sensory): 29 → 10; 3. SF-MPQ (affective): 8 → 1; 4. EQ-5D: 0.356 → 0.751	NR
Kim et al. (2024)	1. VAS (fatigue, cognitive dysfunction); 2. CIS; 3. ChFS (total, physical, mental); 4. K-MoCA; 5. CFQ; 6. EQ-5d-5l; 7. PSQI-K; 8. BDI	1. Fatigue VAS improved within groups, with the highest success rate in the BIT group; 2. No significant between-group difference was observed for cognitive dysfunction VAS; 3. CIS improved significantly in all groups; 4. ChFS showed numerical improvement, without significant between-group difference; 5. K-MoCA improved only in the KOG group (p < 0.05); 6. CFQ improved during follow-up in all groups; 7. PSQI-K improved in the KOG group (p < 0.05); 8. BDI improved in the BIT and KOG groups (p < 0.05)	TG: BIT (33.3%), KOG (6.7%), CBD (26.7%); all mild, none drug-related
Kim and Keum (2015)	NIH-CPSI (total, pain, urinary, quality-of-life)	NIH-CPSI total: 37.6 ± 5.8 → 7.5 ± 3.9 (p < 0.001); pain: 17.5 ± 3.9 → 3.1 ± 2.3 (p < 0.001); urinary: 9.2 ± 1.0 → 1.5 ± 0.9 (p < 0.001); QoL: 10.9 ± 1.4 → 2.8 ± 1.3 (p < 0.001)	NR
Kitahara et al. (2021)	1. MRSA colonization; 2. Febrile infectious diseases; 3. Modified rankin scale (mRS)	1. MRSA colonization: 7.3% (3/41) vs. 25.0% (8/32) (p = 0.0497); 2. Febrile infectious diseases: 17.1% (7/41) vs. 46.9% (15/32) (p = 0.0096); 3. mRS: 4.66 ± 0.88 vs. 5.06 ± 0.44 (p = 0.0329)	NR
Ko et al. (2025)	1. AE & irAE incidence; 2. Fatigue (EORTC-QLQ-c30, FACIT-F, NRS); 3. Muscle loss (SARC-F, body comp., sit-to-stand); 4. Immune markers (PBMC profiling) 5. ORR & DCR	1. AE: 64.29% (9/14) vs. 42.86% (6/14) (p = 0.2556). irAE: 7.14% (1/14) vs. 0% (0/14); 2. Fatigue: improvement trend, Q10 at visit 5 favored BIT (p = 0.0498); 3. SARC-F trend improved vs. placebo (NS); 4. ↓ PD-1+CD8^+^ T cells (p = 0.0492), ↑ NK cells (p = 0.0191); 5. ORR: 16.67% vs. 8.33%; DCR: 41.67% vs. 25.0% (NS)	TG 9/14 (64.29%) vs. CG 6/14 (42.86%); Grade≥3 irAE only in TG: 1 pt (7.14%) with pneumonia; most AEs mild–moderate
Kohno et al. (2021)	1. Rate of VRE negative conversion; 2. Time to negative conversion; 3. Overall survival; 4. Mortality rate	1. VRE negative conversion: 28.2% (11/39) vs. 6.0% (5/83), (p = 0.0014); 2. Shorter time to negative conversion in NG-tube subgroup (p = 0.0485); 3. Improved overall survival with kampo treatment (p = 0.031); 4. Mortality reduced when VRE negative conversion achieved (p = 0.0003)	1 case of hypokalemia (improved with spironolactone)
Lee et al. (2025)	1. Anorexia VAS; 2. SCORAD; 3. DLQI; 4. Topical steroid use; 5. Cytokines (IL-1β, IL-4, IL-17); 6. Gut microbiome changes	1. VAS ↓: week 8 TG 32.33 vs. CG 52.19 (p = 0.0347); 2. SCORAD ↓ only in TG: −11.59 at week 8 (p = 0.0078); 3. DLQI ↓ only in TG at week 8 (p = 0.0113); 4. Topical steroid use lower in TG at week 4 (p = 0.0308); 5. IL-1β ↓ only in TG (p = 0.0057), IL-4 & IL-17 ↓ both groups, more in TG; 6. Microbiome: ↓ gemella. ↑ gemmiger formicilis	TG 10 cases vs. CG 5 cases (NS, p = 0.7602); no serious AEs; 1 fasting glucose elevation in TG judged unrelated
Lee et al. (2021)	1. Progression-free survival (PFS); 2. Tumor response (RECIST); 3. Brain metastasis response; 4. Skin toxicity	1. PFS maintained for >78 months; 2. Tumor size reduced from 3.9 cm → 1.5 cm (61.5% reduction); 3. Brain metastasis: complete disappearance; 4. Skin toxicity: Grade 1→3 rash from gefitinib, improved after dose reduction	No AE associated with BIT reported
Lee et al. (2015)	1. Fatigue (FSS); 2. Quality of life (EORTC QLQ-C30); 3. Blood chemistry & hematology	1. FSS: 36 → 19 after treatment; 2. QOL (global): 50 → 83.3 after treatment; 3. Blood tests: No significant change	NR
Lee et al. (2018)	1. CCMQ (constitution type); 2. SF-36 (quality of life); 3. Flu-like symptom frequency and severity	1. CCMQ: Qi deficiency, blood stasis, and allergy patterns decreased markedly after 2 months; 2. SF-36: general health improved from 10 → 65 (+50%); 3. Flu-like symptoms (fever, dizziness, headache) markedly reduced. Fever subsided, improved appetite and energy	NR
Lee (2017)	1. General weakness; 2. Hair loss recovery; 3. Laboratory findings (CBC, LFT)	1. General weakness improved after 3 months; 2. New hair growth observed after 3 months, black regrowth at 5 months despite ongoing chemotherapy; 3. Hb decreased from 12.9 → 8.0 g/dL, WBC 7880 → 3,470/μL, platelets 320 K → 172 K, indicating no hematologic recovery	NR
Lee et al. (2019)	1. Global assessment scale (GAS) for sweating; 2. GAS for palpitation; 3. Sleep quality; 4. Appetite/weight; 5. Bowel movement	1. GAS for sweating: 100 → 0 after 6 weeks, slight relapse to 20 at week 15; 2. GAS for palpitation: 100 → 0 after 6 weeks; 3. Sleep improved (sleeping time ↑ from 2 to 3 h → 6–7 h); 4. Weight ↑ by 1.5–2 kg; 5. Constipation resolved	NR
Lee et al. (2024)	1. TNSS (r-TNSS & i-TNSS); 2. KARQLQ; 3. Total IgE; 4. Eosinophil count; 5. Overall assessment	1. r-TNSS: High 4.52 ± 1.99 vs. standard 3.97 ± 1.84 vs. Placebo 4.47 ± 1.90 (NS). i-TNSS: High 4.29 ± 2.03 vs. standard 3.53 ± 1.75 vs. Placebo 4.06 ± 1.85 (NS); 2. KARQLQ: All improved. No significant group difference; 3–4. IgE & eosinophils: No significant group differences; 5. Overall assessment: High 2.09 ± 0.84 vs. standard 2.21 ± 0.65 vs. Placebo 2.21 ± 0.59 (NS)	High-dose 11.4% (4/35), standard-dose 11.4% (4/35), Placebo 5.7% (2/35); all mild, none serious
Li et al. (2021a)	1. FSHD evaluation scale; 2. Muscle strength; 3. Weight	1. FSHD score improved from 9 → 6; 2. Muscle strength from 3 to 4/5 → 4–5/5; 3. Weight increased +2.5 kg	NR
Li et al. (2021b)	1. JOMG clinical score (0–1); 2. Anti-AChR antibody (nmol/L)	1. JOMG score improved from 0.9 to 0.2 (overall); 2. AChR-ab decreased from 2.33 to 1.21 nmol/L (overall) 3) each 1 nmol/L ↑ in AChR-ab increased odds of symptom presence by 40% (OR: 1.40) 4) each treatment-time interval ↓ odds of symptoms by 53% (OR: 0.47)	NR
Li et al. (2016)	1. T-cell subsets (CD4, CD8, CD4/CD8); 2. TCM symptom score; 3. Quality of life (EORTC QLQ-C30 domains: function, symptom, global health); 4. AEs	1. CD4 (%) 39.42 ± 5.21 vs. 35.21 ± 9.84. CD8 24.84 ± 5.21 vs. 28.07 ± 7.65. CD4/CD8 1.59 ± 0.23 vs. 1.26 ± 0.28 (P < 0.05); 2. TCM symptom score 5.36 ± 4.98 vs. 12.48 ± 5.21 (P < 0.001); 3. Functional QoL 29.54 ± 3.25 vs. 21.12 ± 2.94. Symptom QoL 30.32 ± 2.97 vs. 19.96 ± 2.25. Global health 28.87 ± 3.64 vs. 20.23 ± 2.45 (all P < 0.001); 4. AE 15% (6/40) vs. 40% (16/40) (P = 0.012)	Leukopenia: 10.0% vs. 20.0%; Hemoglobin↓: 7.5% vs. 15.0%; Platelet↓: 10.0% vs. 17.5%; GI adverse reactions: 15.0% vs. 40.0% (p = 0.012); bone marrow suppression: 17.5% vs. 35.0%
Lu et al. (2021)	1. Total effective rate (Primary); 2. Quality-of-life score; 3. Time to wound healing; 4. Time to pain resolution; 5. Pain index (VAS); 6. Perianal edema score; 7. Anal manometry (anal canal length. Resting pressure. max diastolic. max systolic); 8. Wound exudate score (7 & 14 days)	1. Total effective rate was higher in TG than in CG (p = 0.004); 2. Quality-of-life score was significantly higher in TG (p < 0.001); 3. Time to wound healing was shorter in TG (p < 0.001); 4. Time to pain resolution was shorter in TG (p < 0.001); 5. Pain index was significantly lower in TG (p < 0.001); 6. Perianal edema score was significantly lower in TG (p < 0.001); 7. Anal manometry outcomes were significantly improved in TG (all p ≤ 0.002); 8. Wound exudate score was significantly lower in TG (p < 0.001)	TG 3.3% (2/60) vs. CG 16.7% (10/60), p = 0.013; types included fever, wound exudate, bleeding, perianal edema
Minagawa et al. (2019)	1. Cancer fatigue scale (CFS) score (total & subdomains); 2. Enzalutamide treatment continuation after HET initiation	1. CFS: Improved in 2/3 cases (CFS score decreased). Worsened in 1/3 cases; 2. Enzalutamide continuation duration: 193.0 days (hochuekkito cases) vs. 173.2 days (general fatigue w/o hochuekkito) vs. 265.6 days (no fatigue)	NR
Ni et al. (2021)	1. Sex hormones (testosterone, DHEAS); 2. Ovulation rate; 3. Quality-of-life score; 4. BMI, WHR; 5. LH, FSH, PRL; 6. FBG, FINS, HOMA-IR; 7. Cycle length; 8. Follicle count; 9. Gut microbiota (16S rRNA); 10. Fecal metabolites (metabolomics)	1. Testosterone ↓ (p < 0.001), DHEAS ↓ (p < 0.001); 2. Ovulation ↑ from 33% → 78%; 3. QoL ↑ significantly; 4. BMI ↓, WHR ↓ (p NR); 5. LH ↓ (p < 0.001), FSH ↑ (p < 0.05), PRL ↓ (p < 0.05); 6. FBG ↓ (p < 0.05), FINS ↓ (p < 0.05), HOMA-IR ↓ (NS); 7. Cycle length ↓ (p < 0.001); 8. Follicle count ↓ (p < 0.001); 9. ↑ [Eubacterium]_rectale_group, Escherichia–Shigella, fusicatenibacter (p < 0.05), ↓ megamonas (p < 0.05); 10.106 metabolites changed, 14 KEGG pathways significantly enriched	NR
Oh et al. (2023)	1. Fasting blood sugar (FBS); 2.2-h postprandial glucose (PP2); 3. HbA1c; 4. Liver function (AST, ALT, ALP, GGT); 5. Renal function (BUN, cr, eGFR)	1. FBS: 112.07 ± 18.10 → 108.79 ± 18.47 mg/dL (p = 0.556); 2. PP2: 210.00 ± 60.83 → 165.47 ± 43.90 mg/dL (p = 0.038); 3. HbA1c: 7.92 ± 0.63 → 6.50 ± 1.14 (p = 0.062); 4. AST, ALT, ALP, GGT: No significant change (p > 0.05); 5. BUN, cr, eGFR: No significant change (p > 0.05)	NR
Okabe et al. (2019)	1. Completion rate of 1-year S-1 therapy; 2. Relative dose intensity; 3. AEs; 4. Recurrence-free survival (RFS); 5. Overall survival (OS)	1. Completion rate: TG 54.5% vs. CG 50.9% (p = 0.35); 2. Relative dose intensity: TG 89.2% vs. CG 71.9% (p = 0.33); 3. Grade ≥3 AE: TG 45.5% vs. CG 54.5% (NS); 4. RFS: TG 59.9% vs. CG 70.6% (HR 1.44, p = 0.27); 5. OS: TG 63.9% vs. CG 78.1% (HR 1.68, p = 0.14)	TG 45.5% vs. CG 54.5% Grade ≥3; most common: leukopenia, neutropenia, appetite loss, diarrhea
Okugawa et al. (2024)	1. C-reactive protein (CRP); 2. Albumin; 3. Prognostic Nutritional index (PNI); 4. Body composition by CT (PMI, IMAC, mIMAC); 5. Tumor markers (CEA, CA19-9)	1. CRP: 0.36 → 0.15 mg/dL at 3 months (p < 0.001). metastatic group: 0.49 → 0.28 mg/dL (p = 0.0012); 2. Albumin: No significant change; 3. PNI: No significant change; 4. PMI preserved in CRP-decreased group vs. CRP-increased group (p = 0.028). IMAC & mIMAC NR; 5. Tumor markers: No improvement. CEA and CA19-9 increased	NR
Park et al. (2025)	1. Menstrual pain (NRS); 2. PBAC; 3. EQ-5d-5l; 4. Hemoglobin; 5. Ultrasound (uterine size & MUSA findings)	1. NRS: 7–8 → 1–2; 2. PBAC: >400 → 55; 3. EQ-5d-5l: 0.288 → 0.877; 4. Hemoglobin: 6–8 g/dL → 11 g/dL; 5. Uterine AP length: 8.7 cm → 7.28 cm ultrasound showed decreased adenomyosis area and restoration of normal myometrial tissue	NR
Qian et al. (2024)	1. SNAQ score; 2. EORTC QLQ-C30 domains; 3. CTCAE	1. SNAQ: improved significantly vs. placebo (15.2 ± 1.4 vs. 11.9 ± 1.8, p < 0.001); 2. QoL: multiple EORTC QLQ-C30 domains improved (p < 0.05); 3. CTCAE: Reduced chemotherapy-induced nausea/vomiting, diarrhea, anemia, neutropenia, leukopenia (all p < 0.05). Appetite benefit sustained longer when BIT administered first	Nausea/vomiting 9.1% vs. 62.1% (p < 0.001), diarrhea 12.1% vs. 44.8% (p = 0.004), anemia 15.2% vs. 41.4% (p = 0.021)
Qiu et al. (2024)	1. QMG score; 2. MG-ADL score; 3. Symptom resolution 4. Pyridostigmine withdrawa	1. QMG: 15 → 0; 2. MG-ADL: 11 → 0; 3. Complete clinical remission achieved by month 2, no relapse for 30 months; 4. Pyridostigmine successfully tapered and discontinued	NR
Seo et al. (2019)	1. Daily urine output; 2. Self-voiding urine output; 3. Nelaton catheterization urine volume; 4. CRP level	1. Daily urine output increased after treatment (exact value NR, described as “marked increase”); 2. Self-voiding urine output increased from almost none at baseline to majority of daily urine volume by day 10; 3. Nelaton catheterization volume decreased from daily requirement to near-zero by day 10; 4. CRP decreased from elevated level at admission to normal range (exact value NR)	NR
Takeuchi et al. (2017)	1. AMS (aging males’ symptoms) score; 2. Serum total testosterone (TT) level	1. AMS score improved 55 → 35 after treatment; 2. Serum TT remained unchanged at ∼349.7 ng/dL (baseline vs. post-treatment similar)	NR
Teng et al. (2016)	1. Total effective rate	1. Total effective rate during treatment: TG 97.36% (37/38) vs. CG 96.96% (32/33) (p > 0.05). After follow-up: TG 94.73% (36/38) vs. CG 78.78% (26/33) (p < 0.05)	NR
Terai et al. (2020)	1. Semen volume (mL); 2. Sperm concentration (×10^6^/mL); 3. Sperm motility (%); 4. Total motile sperm count (×10^6^); 5. LH (mIU/mL); 6. FSH (mIU/mL); 7. Testosterone (ng/mL)	1. Semen volume: TG 2.8 ± 1.2 vs. CG 2.7 ± 1.6; 2. Sperm concentration: TG 35.2 ± 35.4 vs. CG 49.1 ± 37.7; 3. Motility: TG 34.0 ± 17.6 vs. CG 31.4 ± 18.3; 4. Total motile sperm count: TG 27.1 ± 29.1 vs. CG 24.1 ± 21.9; 5. LH: TG 4.0 ± 1.8 vs. CG 3.8 ± 1.3; 6. FSH: TG 6.3 ± 3.4 vs. CG 5.4 ± 2.7; 7. Testosterone: TG 4.4 ± 1.0 vs. CG 4.6 ± 1.0	NR
Tokumasu et al. (2025)	1. Fatigue assessment scale (FAS)	1. FAS: 35.9 ± 5.9 → 31.2 ± 9.4 (mean change −4.7.95% CI: 0.5–8.9)	NR
Wang (2016)	1. Total effective rate	1. TG 97.1% (34/35) vs. CG 74.2% (26/35) (p = 0.019)	NR
Wang et al. (2021)	1. KPS score; 2. Serum VEGF (pg/mL); 3. Urine white blood cells (cells/µL); 4. Recurrence rate (6 and 12 months); 5. Adverse reactions	1. KPS: 3 mo TG 83.54 ± 6.37 vs. CG 78.15 ± 10.31 (p = 0.028). 6 mo TG 90.92 ± 9.38 vs. CG 82.47 ± 6.26 (p = 0.034). 12 mo TG 92.23 ± 7.26 vs. CG 88.61 ± 11.32 (p = 0.421); 2. VEGF: 6 mo TG 329.5 ± 121.4 vs. CG 353.7 ± 125.6 (p = 0.042). 12 mo TG 290.3 ± 142.8 vs. CG 331.4 ± 135.2 (p = 0.027). 3 mo NS; 3. Urine WBC: 3 mo TG 20.14 ± 2.09 vs. CG 25.26 ± 4.27 (p = 0.037). 6 and 12 mo NS; 4. Recurrence: 6 mo TG 12.12% (4/33) vs. CG 18.75% (6/32) (p = 0.459). 12 mo TG 18.18% (6/33) vs. CG 25.00% (8/32) (p = 0.504); 5. See safety	Urgent urination: TG 36.36% vs. CG 78.13% (p = 0.043); Nausea/loss of appetite: TG 24.24% vs. CG 50.00% (p = 0.031); low fever: TG 15.15% vs. CG 25.00% (p = 0.321); hematuria: TG 18.18% vs. CG 34.38% (p = 0.137); abnormal urine routine: TG 21.21% vs. CG 46.88% (p = 0.029)
Watanabe and Maeda (2022)	1. Physical activity (walking ex, vigorous activity time); 2. Dietary calorie intake; 3. EQ-VAS; 4. Days to walking milestones	1. Walking ex at 10 weeks: TG 0.03 ± 0.01 vs. CG 0.02 ± 0.01 ex (p = 0.036). Vigorous activity at 8 weeks: TG 1.2 ± 0.7 vs. CG 0.6 ± 0.4 min (p = 0.026); 2. Dietary calories ↑: 10 wks TG 1374 ± 152 vs. CG 1165 ± 233 kcal/day (p = 0.016). Discharge TG 1352 ± 175 vs. CG 1204 ± 253 kcal/day (p = 0.035;. 3. EQ-VAS at 6 weeks: TG 80.4 ± 15.0 vs. CG 64.7 ± 23.9 (p = 0.038); 4. Days to walking with cane shorter in TG (p = 0.042)	TG: 10 AEs in 7 pts; 5 ADRs: Hypokalemia (2), dysuria (1), constipation (1), dry mouth (1). CG: 5 AEs in 5 pts
Wu et al. (2017)	1. Wound healing time (days); 2. Wound flora composition (16S rRNA sequencing)	1. Healing time: BYD 37.50 ± 2.34 vs. positive control 42.60 ± 5.27 (p < 0.05) vs. Negative control 32.16 ± 4.26 (p < 0.05); 2. Flora: BYD ↓ fusobacteria (0.04% vs. 5.29%, p < 0.05) & ↓ cyanobacteria (0.46% vs. 11.88%, p < 0.05) vs. positive control. BYD ↑ bacteroidetes (20.25% vs. 14.15%, p < 0.05) & ↑ proteobacteria (12.98% vs. 11.44%, p < 0.05)	NR
Xu et al. (2023)	1. Clinical success rate; 2. Pathogen eradication rate; 3. CPIS; 4. Mechanical ventilation duration (days); 5.28-day mortality	1. Clinical success: TG 40/83 (48.2%) vs. CG 28/85 (32.9%) (p = 0.044); 2. Pathogen eradication: TG 49/83 (59.0%) vs. CG 33/85 (38.9%) (p = 0.009); 3. CPIS: TG 8.9 ± 1.7 vs. CG 9.6 ± 2.5 (p = 0.003); 4. MV duration: TG 13.7 ± 6.4 days vs. CG 17.2 ± 7.2 days (p = 0.038); 5.28-day mortality: TG 11/83 (13.3%) vs. CG 18/85 (21.2%) (p > 0.05)	Hypoproteinemia: TG 17/83 (20.5%) vs. CG 18/85 (21.2%); no SAE; none related to BIT
Yang (2016)	1. Clinical effective rate; 2. Endoscopic mucosal healing; 3.3-month recurrence rate	1. Clinical effectiveness: Reflux symptoms 43/60 (71.67%) vs. 32/54 (59.26%) (p = 0.007). Esophageal symptoms 40/52 (76.92%) vs. 25/41 (60.98%) (p = 0.036). Extra-esophageal symptoms 21/38 (55.26) vs. 15/30 (50.00) (p = 0.419); 2. Endoscopic mucosal healing: TG showed significantly greater mucosal healing vs. CG (p = 0.031); 3. Recurrence after 3 months: TG 26/64 (40.63%) vs. CG 38/58 (65.52%) (p = 0.026)	TG: Nausea 3, appetite loss3 CG: Nausea 5, vomiting 2, constipation 1
Zhang and Zhu (2015)	1. Vaginal discharge duration (days); 2. Bleeding during scab-shedding (n/N); 3. Complete wound healing time (days)	1. Vaginal discharge: TG 6.4 ± 3.46 days vs. CG 10.8 ± 6.14 days (p < 0.01); 2. Bleeding during scab-shedding: TG 1/30 (3.3%) more-than-menses, 4/30 (13.3%) equal, 25/30 (83.3%) less vs. CG 3/30 (10.0%) more, 8/30 (26.7%) equal, 19/30 (63.3%) less (p < 0.01); 3. Complete wound healing: TG 42 ± 7.75 days vs. CG 63 ± 5.29 days (p < 0.01)	NR
Zhou et al. (2022)	1. Patient global impression of improvement (PGI-I); 2. ICIQ-SF score; 3.72-h urine leakage frequency	1. PGI-I “very much better”: 68.89% at 1 month → 40.00% at 1 year. “no change”: 8.89% → 30.00%; 2. ICIQ-SF: 12.08 ± 5.10 → 2.82 ± 4.53 (1 month) → 4.90 ± 6.21 (1 year) (p < 0.05 vs. baseline. 1-year worse vs. 1-month, p < 0.05); 3.72-h urine leakage: 6.72 ± 6.61 → 1.32 ± 3.72 (1 month) → 3.00 ± 6.16 (1 year) (p < 0.05 vs. baseline. 1-year worse vs. 1-month, p < 0.05)	NR

AE, adverse event; ADR, adverse drug reaction; BIT, Bojungikki-tang; CAT, COPD, assessment test; CBD, Cheonwangbosim-dan; CG, control group; CPIS, clinical pulmonary infection score; CRP, C-reactive protein; CTCAE, common terminology criteria for adverse events; DCR, disease control rate; DLQI, dermatology life quality index; EORTC QLQ-C30, European Organisation for Research and Treatment of Cancer Quality of Life Questionnaire Core 30; EQ-5D, EuroQol 5-Dimension; EQ-VAS, EuroQol visual analogue scale; ICIQ-SF, International Consultation on Incontinence Questionnaire–Short Form; KOG, Kyungok-go; KPS, karnofsky performance status; mMRC, modified Medical Research Council; NR, not reported; NS, not significant; ORR, objective response rate; QOL, quality of life; RECIST, response evaluation criteria in solid tumors; RFS, recurrence-free survival; TG, treatment group; TNSS, total nasal symptom score; VRE, vancomycin-resistant Enterococci.

### 3.5 Safety reporting and adverse events

Safety reporting was uneven across the included studies, and many did not provide detailed AE information (Akita et al., 2019; Chen et al., 2024; Chen et al., 2018; Hamada et al., 2018; Huang, 2015; Huang et al., 2025; Kim et al., 2023; Kim and Keum, 2015; Lee et al., 2021; Lee et al., 2015; Lee et al., 2018; Lee, 2017; Lee et al., 2019; Li et al., 2021a; Li et al., 2021b; Minagawa et al., 2019; Ni et al., 2021; Oh et al., 2023; Okugawa et al., 2024; Park et al., 2025; Qiu et al., 2024; Seo et al., 2019; Takeuchi et al., 2017; Teng et al., 2016; Terai et al., 2020; Tokumasu et al., 2025; Wang, 2016; Wu et al., 2017; Zhang and Zhu, 2015; Zhou et al., 2022). Among studies that reported adverse events (AEs), events were typically mild to moderate, with few reports suggestive of BIT-related toxicity. Transient liver dysfunction was described in one participant and resolved after discontinuation (Edahiro et al., 2022), and mild liver dysfunction of uncertain causality was noted in a COPD study (Hamada et al., 2022). Several controlled studies in oncology and postoperative populations reported lower overall AE rates or fewer treatment-related symptoms in the BIT groups (Hu et al., 2024; Li et al., 2016; Lu et al., 2021; Qian et al., 2024; Wang et al., 2021), while one gastric cancer trial reported comparable rates of grade ≥3 events between groups (Okabe et al., 2019). In infection-related settings, hypokalemia was reported in one case (Kohno et al., 2021), and a hospital-acquired pneumonia trial reported no serious AEs and no events judged related to treatment (Xu et al., 2023). In trials involving allergic rhinitis and atopic dermatitis–associated anorexia, mild events were reported without meaningful between-group differences (Lee et al., 2025; Lee et al., 2024), and a long COVID trial reported only mild AEs with largely stable laboratory parameters (Kim et al., 2024). In addition, a geriatric reflux esophagitis study reported mild gastrointestinal symptoms such as nausea and appetite loss, without evidence of serious treatment-related toxicity (Yang, 2016). A postoperative hip fracture trial recorded several events considered adverse drug reactions, including hypokalemia, dysuria, constipation, and dry mouth (Watanabe and Maeda, 2022). An advanced non-small-cell lung cancer (NSCLC) study conducted alongside atezolizumab reported a numerically higher AE incidence in the BIT group, including one grade ≥3 immune-related pneumonia case (Ko et al., 2025), although most events were mild to moderate and overall differences were not statistically significant (Table 3).

## 4 Discussion

This scoping review mapped 47 clinical studies on BIT published since 2015 and found a broad but uneven evidence landscape across clinical areas. Rather than supporting a single disease-centered indication, the literature concentrates on contexts where symptom burden and recovery are central, with oncology representing the largest cluster of clinical use. This pattern is consistent with recent oncology-focused synthesis work highlighting supportive care, symptom control, and immune-related outcomes as recurring themes in human research on BIT (Cho et al., 2024). Taken together, the evidence base appears to have expanded in scope without a parallel convergence on standardized trial models, formulations, and outcome frameworks, which has implications for both interpretability and future trial prioritization.

The predominance of oncology studies in the present review is clinically meaningful. Among the 13 cancer-related studies, BIT was typically evaluated as an adjunctive intervention across distinct oncology treatment contexts, including chemotherapy or chemoradiotherapy for solid tumors, perioperative recovery, hematologic supportive care, and immune checkpoint inhibitor combination treatment, rather than as a stand-alone antitumor intervention (Edahiro et al., 2022; Hu et al., 2024; Ko et al., 2025; Lee et al., 2021; Lee et al., 2015; Lee, 2017; Lee et al., 2019; Li et al., 2016; Minagawa et al., 2019; Okabe et al., 2019; Okugawa et al., 2024; Qian et al., 2024; Wang et al., 2021). Therefore, this oncology cluster should not be interpreted as a homogeneous clinical category. These treatment contexts imply distinct clinical rationales and outcome priorities: chemotherapy- or chemoradiotherapy-associated studies mainly emphasized appetite, fatigue, nutritional status, quality of life, inflammatory markers, and treatment-related symptoms; perioperative or postoperative studies focused more on recovery trajectories and treatment tolerance; immunotherapy-combination studies incorporated immune response and progression-related outcomes; and the hematologic supportive-care context focused mainly on quality of life and symptom burden. This pattern indicates that BIT has been positioned primarily to address treatment- and disease-associated functional decline, where patient-centered improvements may be clinically relevant even when tumor-control endpoints are not the primary focus or are inconsistently assessed. A similar emphasis is described in broader discussions of Kampo or East Asian botanical drug–based supportive cancer care, where interventions are more often evaluated for symptom burden, functional resilience, and treatment-related adverse effects than for definitive oncologic endpoints such as survival or progression measures (Motoo and Cameron, 2022; Oteki et al., 2016; Okada et al., 2020). The emergence of immune checkpoint inhibitor combination studies suggests a newer research direction, but it remains uncertain whether such approaches will generate clinically decisive evidence (Ko et al., 2025; Cho et al., 2025).

Respiratory indications provide further support for a shared immune–inflammatory axis across disease systems. Respiratory indications accounted for 6 of 47 studies (12.8%). The included studies spanned chronic inflammatory airway disease, infection-related pneumonia, and post-viral syndromes—clinical contexts typically characterized by immune dysregulation and sustained inflammatory stress rather than isolated disturbances in pulmonary mechanics (Hamada et al., 2018; Hamada et al., 2022; Huang et al., 2025; Kim et al., 2024; Tokumasu et al., 2025, Xu et al., 2023). Accordingly, several studies incorporated inflammatory markers or immune profiles alongside conventional respiratory assessments. Outcome evaluation also extended beyond spirometric indices to include broader functional measures such as dyspnea, activity, and fatigue trajectories, particularly in post-viral recovery contexts (Hamada et al., 2018; Xu et al., 2023; Tokumasu et al., 2025). Similar to oncology, this pattern suggests that BIT has been investigated less as a disease-specific pulmonary modifier and more as a regulator of host immune balance during periods of inflammatory burden and physiologic vulnerability.

Beyond oncology and respiratory disease, a comparable recovery phenotype was evident across postoperative and convalescent settings. In the hip-fracture RCT in older adults, BIT was evaluated during postoperative rehabilitation and was associated with improvements in physical activity and appetite (Watanabe and Maeda, 2022). Similarly, the 2025 exploratory pilot in post-COVID-19 condition tracked fatigue trajectories over an 8-week treatment period (Tokumasu et al., 2025). This endpoint profile aligns with broader discussions of traditional botanical preparations in supportive care, where symptom burden, nutritional intake, and functional resilience are commonly prioritized, particularly for fatigue- and anorexia-related domains (Motoo and Cameron, 2022; Zedler et al., 2024). Recent reviews of post-COVID fatigue management likewise highlight functional recovery and persistent symptom burden as central targets, mirroring the tendency of respiratory and post-viral studies in this review to converge on fatigue and activity outcomes (Chen et al., 2025). Taken together, these patterns suggest that despite diagnostic diversity, the included conditions share a common clinical expression of reduced functional reserve and delayed recovery, positioning BIT as a potential resilience-enhancing intervention across physiologic vulnerability states. This recovery-oriented framing should be interpreted as an authors’ interpretive synthesis based on recurring populations, outcomes, and clinical contexts, rather than as an empirically confirmed mechanism or treatment effect.

Despite the growth of controlled trials, the included literature showed marked heterogeneity in how BIT was delivered and evaluated, limiting cross-study interpretability. BIT was used as monotherapy in 26 of 47 studies (55.3%), while 21 studies (44.7%) assessed it as an adjunct to conventional care, rehabilitation, pharmacotherapy, or other integrative interventions. Formulation varied substantially: granule preparations were used in 22 studies (46.8%) (Akita et al., 2019; Edahiro et al., 2022; Hamada et al., 2018; Hamada et al., 2022; Huang et al., 2025; Kim et al., 2024; Kitahara et al., 2021; Ko et al., 2025; Kohno et al., 2021; Lee et al., 2025; Lee et al., 2015; Lee, 2017; Lee et al., 2024; Minagawa et al., 2019; Oh et al., 2023; Okabe et al., 2019; Okugawa et al., 2024; Takeuchi et al., 2017; Terai et al., 2020; Tokumasu et al., 2025; Watanabe and Maeda, 2022; Wu et al., 2017) and decoctions in 20 studies (42.6%) (Chen et al., 2024; Chen et al., 2018; Hu et al., 2024; Huang, 2015; Kim et al., 2023; Lee et al., 2021; Li et al., 2021a; Li et al., 2021b; Li et al., 2016; Lu et al., 2021; Ni et al., 2021; Park et al., 2025; Qiu et al., 2024; Seo et al., 2019; Teng et al., 2016; Wang, 2016; Wang et al., 2021; Xu et al., 2023; Zhang and Zhu, 2015; Zhou et al., 2022), together accounting for 89.4% of interventions. However, these categories may understate clinically important differences, because granule-based studies more often used fixed commercial products, whereas decoction-based studies frequently employed modified prescriptions with additional botanical drugs or flexible adjustment. Whereas granule-based studies more often used fixed commercial products.

This distinction is clinically relevant because standardized granules and highly modified decoctions may represent different pharmacological exposures, dose–exposure assumptions, and levels of reproducibility. In addition, formulation type appeared to overlap with country of origin and study design, as Japanese studies more often used fixed granule products whereas several Chinese studies used modified decoction-based prescriptions, and South Korean studies included a larger proportion of case-based reports. These differences may reflect distinct prescribing traditions, regulatory environments, reimbursement systems, and research infrastructures across East Asian healthcare settings, and may confound direct interpretation of outcomes across formulation categories. Therefore, geographic clustering should be considered when interpreting formulation choice, study design, outcome selection, AE reporting, and the generalizability of the mapped evidence. Comparator strategies were similarly diverse, spanning placebo-controlled designs, usual-care comparisons, active co-interventions, and uncontrolled observations. As a result, identical preparation labels often represented different compositional and dosing realities, and findings were difficult to contrast directly even within the same disease system. Under these conditions, the evidence is best interpreted as mapping where and how BIT has been studied rather than as supporting a single transferable estimate of effect across indications.

Outcome reporting varied widely across the included studies, reflecting both the broad range of indications and the lack of a shared measurement framework. Many trials relied on symptom-centered patient-reported outcomes and condition-specific clinical scores (Kim et al., 2023; Kim and Keum, 2015; Chen et al., 2024; Chen et al., 2018; Zhou et al., 2022; Seo et al., 2019), often paired with generic or disease-specific quality-of-life instruments (Edahiro et al., 2022; Hamada et al., 2018; Hu et al., 2024; Kim et al., 2023; Kim et al., 2024; Lee et al., 2025; Lee et al., 2015; Lee et al., 2018; Lee et al., 2024; Li et al., 2016; Park et al., 2025; Qian et al., 2024; Watanabe and Maeda, 2022). Functional performance endpoints were common in respiratory and postoperative settings (Hamada et al., 2018; Hamada et al., 2022; Watanabe and Maeda, 2022), whereas neurologic and neuromuscular studies more often used disease-specific functional scales (Kitahara et al., 2021; Li et al., 2021a; Li et al., 2021b; Qiu et al., 2024). Biomarkers were frequently incorporated, spanning inflammatory markers, nutritional indices, immune profiles, and metabolic or endocrine parameters (Akita et al., 2019; Hu et al., 2024; Huang, 2015; Ko et al., 2025; Lee et al., 2025; Li et al., 2016; Oh et al., 2023; Okugawa et al., 2024; Takeuchi et al., 2017; Terai et al., 2020), with a smaller subset reporting microbiome-related measures (Huang et al., 2025; Lee et al., 2025; Ni et al., 2021; Wu et al., 2017). This breadth suits a scoping review approach (Arksey and O’Malley, 2005; Tricco et al., 2018; Peters et al., 2020), but substantial within-category heterogeneity in instruments, follow-up windows, and analytic handling limits comparability. Recent reporting guidance emphasizes that outcomes should be defined explicitly with prespecified time points and transparent analytic rules to support interpretation and synthesis, yet these elements were inconsistently described in the included literature (Butcher et al., 2022). As a result, outcome themes can be mapped, but effects remain difficult to synthesize across studies.

Safety reporting was a clear weak point in the included literature. Many studies did not provide detailed adverse-event information, and structured approaches to causality assessment were rarely described (Akita et al., 2019; Chen et al., 2024; Chen et al., 2018; Hamada et al., 2018; Huang, 2015; Huang et al., 2025; Kim et al., 2023; Kim and Keum, 2015; Lee et al., 2021; Lee et al., 2015; Lee et al., 2018; Lee, 2017; Lee et al., 2019; Li et al., 2021a; Li et al., 2021b; Minagawa et al., 2019; Ni et al., 2021; Oh et al., 2023; Okugawa et al., 2024; Park et al., 2025; Qiu et al., 2024; Seo et al., 2019; Takeuchi et al., 2017; Teng et al., 2016; Terai et al., 2020; Tokumasu et al., 2025; Wang, 2016; Wu et al., 2017; Zhang and Zhu, 2015; Zhou et al., 2022). Among the 23 RCTs, 13 studies provided some structured AE information, whereas 10 studies did not provide detailed AE information. Incomplete reporting of botanical drug composition, product characteristics, processing and extraction information, concomitant medications, AEs, and interaction-related safety data limited the direct interpretation and comparability of safety findings across studies. When AEs were reported, they were usually mild to moderate and serious events appeared uncommon, but this impression is constrained by incomplete documentation rather than robust negative evidence. Therefore, the current safety evidence remains insufficient, and underreporting may have biased the apparent tolerability of BIT. Current trial standards, including CONSORT Harms 2022, increasingly expect transparent harms reporting, including how events are defined, actively collected, graded, and attributed; without those details, tolerability signals may be underestimated and potential drug–botanical preparation interaction risks can be difficult to evaluate, particularly with complex co-medication exposure (Junqueira et al., 2023). Reporting assessments in Chinese botanical drug trials similarly suggest persistent shortcomings in intervention and safety reporting despite dedicated guideline extensions (Wang et al., 2024). Future BIT trials should therefore document concomitant medications, monitoring schedules, discontinuations, adverse-event attribution, and interaction-related safety data more systematically, particularly in oncology and multimorbidity contexts.

Several implications emerge from this mapped evidence. From a methodological perspective, differences in intervention delivery, comparator selection, outcome definition, and safety reporting limit cross-study synthesis and indicate that the current literature does not yet support a single transferable estimate of effect across disease categories. Because this was a scoping review, we did not conduct a formal risk-of-bias assessment, certainty-of-evidence appraisal, or structured reporting quality assessment, which should be regarded as an important limitation, particularly because nearly half of the included studies were RCTs. In addition, the lack of explicit primary and secondary outcome designation in many studies limited the ability to construct a structured outcome hierarchy for future core outcome development. Although all included studies were published in peer-reviewed journals, variation in journal visibility and reporting standards may have influenced the completeness of methodological and safety reporting. Therefore, the findings should be interpreted as a map of the clinical evidence landscape rather than as a judgment on the reliability, effectiveness, or safety of BIT. These features are consistent with a research field that has expanded across multiple clinical areas without a parallel convergence on unified trial models (Zhong et al., 2010). Nevertheless, despite these methodological constraints, the literature reveals a set of recurring clinical patterns. Across oncology and respiratory disease in particular, studies repeatedly situate BIT within contexts characterized by immune–inflammatory burden and reduced physiologic reserve. Fatigue, appetite disturbance, and quality-of-life impairment appear consistently as dominant outcome domains, suggesting that many of the included populations share a common clinical phenotype of physiologic vulnerability and delayed recovery. In this sense, oncology supportive care, respiratory and post-viral conditions, and postoperative recovery contexts appear to converge on a similar therapeutic framing.

Taken together, these repeated patterns suggest that although the evidence base remains methodologically heterogeneous, BIT is being positioned across multiple disease systems as a recovery-oriented intervention that may modulate immune–inflammatory stress and support restoration of functional resilience. Future studies would therefore benefit from tighter specification of formulation, clearer differentiation between fixed commercial products and modified prescriptions, and adoption of shared recovery-oriented outcomes such as fatigue, appetite, physical activity, and quality of life. Standardized harms reporting, clear separation of treatment duration and post-treatment follow-up, and prespecified time points would further strengthen interpretability. Equally important, future studies should avoid treating BIT as a uniform intervention unless botanical drug composition, product characteristics, processing, extraction, and dosage are sufficiently comparable. A strategic shift toward fewer, larger, and methodologically aligned trials within these recurring clinical clusters may clarify the potential recovery-oriented role of BIT more effectively than continued expansion into rarely studied or methodologically isolated indications.

## 5 Conclusion

This scoping review identified 47 clinical studies on BIT published since 2015 and found that the current evidence base is concentrated in supportive-care contexts rather than in a single disease-specific indication. Cancer-related conditions were the largest clinical cluster, and nearly half of all included studies were RCTs, but substantial heterogeneity remained in formulation, comparator strategy, treatment duration, and outcome selection. Across indications, the most consistent research theme was not disease modification itself, but improvement in fatigue, appetite, functional recovery, quality of life, and treatment tolerance. These findings suggest that, based on research volume and thematic convergence rather than demonstrated efficacy, the most productive next step is not broader exploratory use across many unrelated conditions, but more rigorous and standardized trials in a limited number of recurrently studied clinical scenarios, particularly oncology supportive care and other recovery-focused settings. However, the frequency of investigation should not be interpreted as evidence of benefit, and future studies are needed to determine whether BIT provides clinically meaningful effects in these contexts.

## Data Availability

The original contributions presented in the study are included in the article/Supplementary Material, further inquiries can be directed to the corresponding author.
