# Supplementary material for: Current clinical applications and clinical evidence of Bojungikki-tang, a traditional East Asian multi-botanical preparation: a scoping review
Source: Front Pharmacol. 2026 Jul 10;17:1831561. doi: 10.3389/fphar.2026.1831561 (PMC13396291; doi:10.3389/fphar.2026.1831561)
Supplement: Supplementary file 1 [file Table1.docx]

Supplementary Material

**Supplementary Table S1**. Search Strategies Applied Across Databases

| Database | Search Strategy | Records Retrieved |
| --- | --- | --- |
| PubMed | ("Buzhongyiqi"[tiab] OR "Buzhong Yiqi"[tiab] OR "Bu Zhong Yi Qi"[tiab] OR "Buzhong Yiqi Tang"[tiab] OR "Hochuekkito"[tiab] OR "Hochu-ekkito"[tiab] OR "Hochu-ekki-to"[tiab] OR "Hochu-ekki-to TJ-41"[tiab] OR "TJ 41"[tiab] OR "TJ-41"[tiab] OR "Bojungikgitang"[tiab] OR "Bojungikgi"[tiab] OR "Bojungikki-tang"[tiab]) | **222** |
| EMBASE | ('buzhongyiqi':ti,ab OR 'buzhong yiqi':ti,ab OR 'bu zhong yi qi':ti,ab OR 'buzhong yiqi tang':ti,ab OR 'hochuekkito':ti,ab OR 'hochu-ekkito':ti,ab OR 'hochu-ekki-to':ti,ab OR 'hochuekkito tj-41':ti,ab OR 'hochu-ekkito tj-41':ti,ab OR 'tj 41':ti,ab OR 'tj-41':ti,ab OR 'tj41':ti,ab OR 'bojungikgitang':ti,ab OR 'bojungikgi':ti,ab OR 'bojungikki-tang':ti,ab OR 'bojungikki':ti,ab) | **302** |
| CENTRAL | ("Buzhongyiqi" OR "Buzhong Yiqi" OR "Bu Zhong Yi Qi" OR "Buzhong Yiqi Tang" OR Hochuekkito OR "Hochu ekkito" OR "Hochu ekki to" OR "TJ 41" OR "TJ41" OR "Bojungikgitang" OR "Bojungikki tang" OR "Bojungikki" OR "Bojungikgi") | **127** |
| OASIS | 보중익기탕\|補中益氣 | **16** |

**Supplementary Table S2.** Taxonomic validation of medicinal materials used in Bojungikki-tang and modified formulations

| **Study** | **Description** | **Composition** |
| --- | --- | --- |
| Akita 2019 | Bojungikki-tang | Panax ginseng C.A.Mey. [Araliaceae; Ginseng radix], Atractylodes spp. [Asteraceae; Atractylodis rhizoma], Astragalus membranaceus (Fisch.) Bunge [Fabaceae; Astragali radix], Angelica spp. [Apiaceae; Angelicae radix], Ziziphus jujuba Mill. [Rhamnaceae; Ziziphi fructus], Bupleurum spp. [Apiaceae; Bupleuri radix], Glycyrrhiza uralensis Fisch. ex DC. [Fabaceae; Glycyrrhizae radix], Zingiber officinale Roscoe [Zingiberaceae; Zingiberis rhizoma], Actaea spp. [Ranunculaceae; Cimicifugae rhizoma], Citrus spp. [Rutaceae; Aurantii nobilis pericarpium] |
| Chen 2024 | Bojungikki-tang + biofeedback electrical stimulation | Astragalus membranaceus (Fisch.) Bunge [Fabaceae; Astragali radix], Angelica sinensis (Oliv.) Diels [Apiaceae; Angelicae sinensis radix], Leonurus japonicus Houtt. [Lamiaceae; Leonuri herba], Codonopsis pilosula (Franch.) Nannf. [Campanulaceae; Codonopsis radix], Dipsacus asper Wall. ex C.B.Clarke [Caprifoliaceae; Dipsaci radix], Atractylodes macrocephala Koidz. [Asteraceae; Atractylodis macrocephalae rhizoma], Actaea heracleifolia (Kom.) J.Compton [Ranunculaceae; Cimicifugae rhizoma], Bupleurum chinense DC. [Apiaceae; Bupleuri radix], Citrus reticulata Blanco [Rutaceae; Citri reticulatae pericarpium], Ligusticum striatum DC. [Apiaceae; Chuanxiong rhizoma], Amomum villosum Lour. [Zingiberaceae; Amomi fructus], Terminalia chebula Retz. [Combretaceae; Chebulae fructus], Cornus officinalis Siebold & Zucc. [Cornaceae; Corni fructus], Glycyrrhiza uralensis Fisch. ex DC. [Fabaceae; Glycyrrhizae radix praeparata] |
| Chen 2018 | Modified Bojungikki-tang + pelvic floor muscle exercise + biofeedback + electrical stimulation | Codonopsis pilosula (Franch.) Nannf. [Campanulaceae; Codonopsis radix], Atractylodes macrocephala Koidz. [Asteraceae; Atractylodis macrocephalae rhizoma], Actaea heracleifolia (Kom.) J.Compton [Ranunculaceae; Cimicifugae rhizoma], Astragalus membranaceus (Fisch.) Bunge [Fabaceae; Astragali radix], Angelica sinensis (Oliv.) Diels [Apiaceae; Angelicae sinensis radix], Citrus reticulata Blanco [Rutaceae; Citri reticulatae pericarpium], Glycyrrhiza uralensis Fisch. ex DC. [Fabaceae; Glycyrrhizae radix praeparata], Leonurus japonicus Houtt. [Lamiaceae; Leonuri herba], Ligusticum striatum DC. [Apiaceae; Chuanxiong rhizoma], Dipsacus asper Wall. ex C.B.Clarke [Caprifoliaceae; Dipsaci radix], Cornus officinalis Siebold & Zucc. [Cornaceae; Corni fructus], Terminalia chebula Retz. [Combretaceae; Chebulae fructus], Alpinia oxyphylla Miq. [Zingiberaceae; Alpiniae oxyphyllae fructus] |
| Edahiro 2022 | Bojungikki-tang | Astragalus membranaceus (Fisch.) Bunge [Fabaceae; Astragali radix], Atractylodes lancea (Thunb.) DC. [Asteraceae; Atractylodis lanceae rhizoma], Panax ginseng C.A.Mey. [Araliaceae; Ginseng radix], Angelica acutiloba (Siebold & Zucc.) Kitag. [Apiaceae; Angelicae acutilobae radix], Bupleurum falcatum L. [Apiaceae; Bupleuri radix], Ziziphus jujuba Mill. [Rhamnaceae; Ziziphi fructus], Citrus unshiu Marcow. [Rutaceae; Citri unshius pericarpium], Glycyrrhiza uralensis Fisch. ex DC. [Fabaceae; Glycyrrhizae radix], Actaea heracleifolia (Kom.) J.Compton [Ranunculaceae; Cimicifugae rhizoma], Zingiber officinale Roscoe [Zingiberaceae; Zingiberis rhizoma] |
| Hamada 2018 | Bojungikki-tang + pulmonary rehabilitation | Astragalus membranaceus (Fisch.) Bunge [Fabaceae; Astragali radix], Atractylodes lancea (Thunb.) DC. [Asteraceae; Atractylodis lanceae rhizoma], Panax ginseng C.A.Mey. [Araliaceae; Ginseng radix], Angelica acutiloba (Siebold & Zucc.) Kitag. [Apiaceae; Angelicae radix], Bupleurum falcatum L. [Apiaceae; Bupleuri radix], Ziziphus jujuba Mill. [Rhamnaceae; Ziziphi fructus], Citrus unshiu Marcow. [Rutaceae; Aurantii nobilis pericarpium], Glycyrrhiza uralensis Fisch. ex DC. [Fabaceae; Glycyrrhizae radix], Actaea spp. [Ranunculaceae; Cimicifugae rhizoma], Zingiber officinale Roscoe [Zingiberaceae; Zingiberis rhizoma] |
| Hamada 2022 | Bojungikki-tang + pulmonary rehabilitation | Astragalus membranaceus (Fisch.) Bunge [Fabaceae; Astragali radix], Atractylodes lancea (Thunb.) DC. [Asteraceae; Atractylodis lanceae rhizoma], Panax ginseng C.A.Mey. [Araliaceae; Ginseng radix], Angelica acutiloba (Siebold & Zucc.) Kitag. [Apiaceae; Angelicae radix], Bupleurum falcatum L. [Apiaceae; Bupleuri radix], Ziziphus jujuba Mill. [Rhamnaceae; Ziziphi fructus], Citrus unshiu Marcow. [Rutaceae; Aurantii nobilis pericarpium], Glycyrrhiza uralensis Fisch. ex DC. [Fabaceae; Glycyrrhizae radix], Actaea spp. [Ranunculaceae; Cimicifugae rhizoma], Zingiber officinale Roscoe [Zingiberaceae; Zingiberis rhizoma] |
| Hu 2024 | Bojungikki-tang + conventional rehabilitation after laparoscopic surgery | Angelica sinensis (Oliv.) Diels [Apiaceae; Angelicae sinensis radix], Dioscorea polystachya Turcz. [Dioscoreaceae; Dioscoreae rhizoma], Citrus reticulata Blanco [Rutaceae; Aurantii nobilis pericarpium], Bupleurum chinense DC. [Apiaceae; Bupleuri radix], Glycyrrhiza uralensis Fisch. ex DC. [Fabaceae; Glycyrrhizae radix], Panax ginseng C.A.Mey. [Araliaceae; Ginseng radix], Actaea heracleifolia (Kom.) J.Compton [Ranunculaceae; Cimicifugae rhizoma], Atractylodes macrocephala Koidz. [Asteraceae; Atractylodis macrocephalae rhizoma], Amomum villosum Lour. [Zingiberaceae; Amomi fructus], Poria cocos (Schw.) Wolf [Polyporaceae; Poria], Astragalus membranaceus (Fisch.) Bunge [Fabaceae; Astragali radix] |
| Huang 2015 | Modified Bojungikki-tang + metformin + acarbose | Angelica sinensis (Oliv.) Diels [Apiaceae; Angelicae sinensis radix], Atractylodes macrocephala Koidz. [Asteraceae; Atractylodis macrocephalae rhizoma], Lycium barbarum L. [Solanaceae; Lycii fructus], Panax ginseng C.A.Mey. [Araliaceae; Ginseng radix], Rehmannia glutinosa (Gaertn.) DC. [Orobanchaceae; Rehmanniae radix], Poria cocos (Schw.) Wolf [Polyporaceae; Poria], Astragalus membranaceus (Fisch.) Bunge [Fabaceae; Astragali radix], Bupleurum chinense DC. [Apiaceae; Bupleuri radix], Actaea heracleifolia (Kom.) J.Compton [Ranunculaceae; Cimicifugae rhizoma], Citrus reticulata Blanco [Rutaceae; Citri reticulatae pericarpium] |
| Huang 2025 | Bojungikki-tang | NR |
| Kim 2023 | Modified Bojungikki-tang + acupuncture + pharmacopuncture | Panax ginseng C.A.Mey. [Araliaceae; Ginseng radix], Astragalus membranaceus (Fisch.) Bunge [Fabaceae; Astragali radix], Atractylodes macrocephala Koidz. [Asteraceae; Atractylodis rhizoma alba], Angelica gigas Nakai [Apiaceae; Angelicae gigantis radix], Citrus reticulata Blanco [Rutaceae; Aurantii nobilis pericarpium], Glycyrrhiza uralensis Fisch. ex DC. [Fabaceae; Glycyrrhizae radix], Perilla frutescens (L.) Britton [Lamiaceae; Perillae herba], Agastache rugosa (Fisch. & C.A.Mey.) Kuntze [Lamiaceae; Agastachis herba], Zingiber officinale Roscoe [Zingiberaceae; Zingiberis rhizoma], Ziziphus jujuba Mill. [Rhamnaceae; Ziziphi fructus], Ephedra sinica Stapf [Ephedraceae; Ephedrae herba], Aconitum carmichaelii Debeaux [Ranunculaceae; Aconiti lateralis radix], Asarum sieboldii Miq. [Aristolochiaceae; Asiasari radix] |
| Kim 2024 | Bojungikki-tang | Panax ginseng C.A.Mey. [Araliaceae; Ginseng radix], Atractylodes japonica Koidz. [Asteraceae; Atractylodis rhizoma], Astragalus membranaceus (Fisch.) Bunge [Fabaceae; Astragali radix], Angelica gigas Nakai [Apiaceae; Angelicae gigantis radix], Ziziphus jujuba Mill. [Rhamnaceae; Ziziphi fructus], Bupleurum falcatum L. [Apiaceae; Bupleuri radix], Citrus unshiu Marcow. [Rutaceae; Citri unshius pericarpium], Glycyrrhiza uralensis Fisch. ex DC. [Fabaceae; Glycyrrhizae radix], Actaea heracleifolia (Kom.) J.Compton [Ranunculaceae; Cimicifugae rhizoma], Zingiber officinale Roscoe [Zingiberaceae; Zingiberis rhizoma] |
| Kim 2015 | Modified Bojungikki-tang | Astragalus membranaceus (Fisch.) Bunge [Fabaceae; Astragali radix], Panax ginseng C.A.Mey. [Araliaceae; Ginseng radix], Glycyrrhiza uralensis Fisch. ex DC. [Fabaceae; Glycyrrhizae radix], Atractylodes macrocephala Koidz. [Asteraceae; Atractylodis macrocephalae rhizoma], Fraxinus rhynchophylla Hance [Oleaceae; Fraxini cortex], Angelica gigas Nakai [Apiaceae; Angelicae gigantis radix], Actaea spp. [Ranunculaceae; Cimicifugae rhizoma], Bupleurum falcatum L. [Apiaceae; Bupleuri radix], Massa Medicata Fermentata [fermented preparation; species composition NR], Hordeum vulgare L. [Poaceae; Hordei fructus germinatus], Crataegus pinnatifida Bunge [Rosaceae; Crataegi fructus] |
| Kitahara 2021 | Bojungikki-tang | Astragalus membranaceus (Fisch.) Bunge [Fabaceae; Astragali radix], Atractylodes japonica Koidz. [Asteraceae; Atractylodis rhizoma], Panax ginseng C.A.Mey. [Araliaceae; Ginseng radix], Angelica acutiloba (Siebold & Zucc.) Kitag. [Apiaceae; Angelicae acutilobae radix], Bupleurum falcatum L. [Apiaceae; Bupleuri radix], Ziziphus jujuba Mill. [Rhamnaceae; Ziziphi fructus], Citrus unshiu Marcow. [Rutaceae; Citri unshius pericarpium], Glycyrrhiza uralensis Fisch. ex DC. [Fabaceae; Glycyrrhizae radix], Actaea simplex (DC.) Wormsk. ex Prantl [Ranunculaceae; Cimicifugae rhizoma], Zingiber officinale Roscoe [Zingiberaceae; Zingiberis rhizoma] |
| Ko 2025 | Bojungikki-tang + atezolizumab | Panax ginseng C.A.Mey. [Araliaceae; Ginseng radix], Atractylodes macrocephala Koidz. [Asteraceae; Atractylodis rhizoma alba], Astragalus membranaceus (Fisch.) Bunge [Fabaceae; Astragali radix], Angelica gigas Nakai [Apiaceae; Angelicae gigantis radix], Ziziphus jujuba Mill. [Rhamnaceae; Ziziphi fructus], Bupleurum falcatum L. [Apiaceae; Bupleuri radix], Citrus unshiu Marcow. [Rutaceae; Citri unshius pericarpium], Actaea spp. [Ranunculaceae; Cimicifugae rhizoma], Zingiber officinale Roscoe [Zingiberaceae; Zingiberis rhizoma recens], Glycyrrhiza uralensis Fisch. ex DC. [Fabaceae; Glycyrrhizae radix et rhizoma] |
| Kohno 2021 | Bojungikki-tang | Astragalus membranaceus (Fisch.) Bunge [Fabaceae; Astragali radix], Atractylodes lancea (Thunb.) DC. [Asteraceae; Atractylodis rhizoma], Panax ginseng C.A.Mey. [Araliaceae; Ginseng radix], Angelica acutiloba (Siebold & Zucc.) Kitag. [Apiaceae; Angelicae acutilobae radix], Bupleurum falcatum L. [Apiaceae; Bupleuri radix], Ziziphus jujuba Mill. [Rhamnaceae; Ziziphi fructus], Citrus unshiu Marcow. [Rutaceae; Citri unshius pericarpium], Glycyrrhiza uralensis Fisch. ex DC. [Fabaceae; Glycyrrhizae radix], Actaea simplex (DC.) Wormsk. ex Prantl [Ranunculaceae; Cimicifugae rhizoma], Zingiber officinale Roscoe [Zingiberaceae; Zingiberis rhizoma] |
| Lee 2025 | Bojungikki-tang | Panax ginseng C.A.Mey. [Araliaceae; Ginseng radix], Atractylodes japonica Koidz. [Asteraceae; Atractylodis rhizoma], Astragalus membranaceus (Fisch.) Bunge [Fabaceae; Astragali radix], Angelica gigas Nakai [Apiaceae; Angelicae gigantis radix], Ziziphus jujuba Mill. [Rhamnaceae; Ziziphi fructus], Bupleurum falcatum L. [Apiaceae; Bupleuri radix], Citrus unshiu Marcow. [Rutaceae; Citri unshius pericarpium], Glycyrrhiza uralensis Fisch. ex DC. [Fabaceae; Glycyrrhizae radix], Actaea heracleifolia (Kom.) J.Compton [Ranunculaceae; Cimicifugae rhizoma], Zingiber officinale Roscoe [Zingiberaceae; Zingiberis rhizoma] |
| Lee 2021 | Gefitinib + modified Bojungikki-tang | Panax ginseng C.A.Mey. [Araliaceae; Ginseng radix], Astragalus membranaceus (Fisch.) Bunge [Fabaceae; Astragali radix], Liriope platyphylla F.T.Wang & T.Tang [Asparagaceae; Liriopes radix], Magnolia obovata Thunb. [Magnoliaceae; Magnoliae cortex], Zingiber officinale Roscoe [Zingiberaceae; Zingiberis rhizoma], Atractylodes japonica Koidz. [Asteraceae; Atractylodis rhizoma], Angelica gigas Nakai [Apiaceae; Angelicae gigantis radix], Citrus reticulata Blanco [Rutaceae; Citri reticulatae pericarpium], Glycyrrhiza uralensis Fisch. ex DC. [Fabaceae; Glycyrrhizae radix], Pinellia ternata (Thunb.) Makino [Araceae; Pinelliae tuber], Ziziphus jujuba Mill. [Rhamnaceae; Ziziphi fructus], Cervus nippon Temminck [Cervidae; Cervi cornu], Agastache rugosa (Fisch. & C.A.Mey.) Kuntze [Lamiaceae; Agastachis herba], Perilla frutescens (L.) Britton [Lamiaceae; Perillae herba] |
| Lee 2015 | Bojungikki-tang | NR |
| Lee 2018 | Modified Bojungikki-tang combined with Salvia miltiorrhiza, Ligusticum chuanxiong, Angelica dahurica, and Polygonum multiflorum | Astragalus membranaceus (Fisch.) Bunge [Fabaceae; Astragali radix], Atractylodes spp. [Asteraceae; Atractylodis rhizoma], Panax ginseng C.A.Mey. [Araliaceae; Ginseng radix], Angelica spp. [Apiaceae; Angelicae radix], Bupleurum spp. [Apiaceae; Bupleuri radix], Ziziphus jujuba Mill. [Rhamnaceae; Ziziphi fructus], Citrus spp. [Rutaceae; Aurantii nobilis pericarpium], Glycyrrhiza uralensis Fisch. ex DC. [Fabaceae; Glycyrrhizae radix], Actaea spp. [Ranunculaceae; Cimicifugae rhizoma], Zingiber officinale Roscoe [Zingiberaceae; Zingiberis rhizoma], Salvia miltiorrhiza Bunge [Lamiaceae; Salviae miltiorrhizae radix et rhizoma], Ligusticum striatum DC. [Apiaceae; Chuanxiong rhizoma], Angelica dahurica (Hoffm.) Benth. & Hook.f. ex Franch. & Sav. [Apiaceae; Angelicae dahuricae radix], Polygonum multiflorum Thunb. [Polygonaceae; Polygoni multiflori radix] |
| Lee 2017 | Bojungikki-tang + allergen-removed Rhus verniciflua extract | Astragalus membranaceus (Fisch.) Bunge [Fabaceae; Astragali radix], Atractylodes spp. [Asteraceae; Atractylodis rhizoma], Panax ginseng C.A.Mey. [Araliaceae; Ginseng radix], Angelica spp. [Apiaceae; Angelicae radix], Bupleurum spp. [Apiaceae; Bupleuri radix], Ziziphus jujuba Mill. [Rhamnaceae; Ziziphi fructus], Citrus spp. [Rutaceae; Aurantii nobilis pericarpium], Glycyrrhiza uralensis Fisch. ex DC. [Fabaceae; Glycyrrhizae radix], Actaea spp. [Ranunculaceae; Cimicifugae rhizoma], Zingiber officinale Roscoe [Zingiberaceae; Zingiberis rhizoma], Toxicodendron vernicifluum (Stokes) F.A.Barkley [Anacardiaceae; allergen-removed Rhus verniciflua extract] |
| Lee 2019 | Modified Bojungikki-tang + acupuncture | Panax ginseng C.A.Mey. [Araliaceae; Ginseng radix], Astragalus membranaceus (Fisch.) Bunge [Fabaceae; Astragali radix], Zingiber officinale Roscoe [Zingiberaceae; Zingiberis rhizoma crudus], Glycyrrhiza uralensis Fisch. ex DC. [Fabaceae; Glycyrrhizae radix], Atractylodes macrocephala Koidz. [Asteraceae; Atractylodis rhizoma alba], Angelica gigas Nakai [Apiaceae; Angelicae gigantis radix], Citrus reticulata Blanco [Rutaceae; Citri pericarpium], Ziziphus jujuba Mill. [Rhamnaceae; Jujubae fructus], Amomum villosum Lour. [Zingiberaceae; Amomi fructus], Amomum kravanh Pierre ex Gagnep. [Zingiberaceae; Amomi rotundus fructus], Alpinia oxyphylla Miq. [Zingiberaceae; Alpiniae oxyphyllae fructus], Agastache rugosa (Fisch. & C.A.Mey.) Kuntze [Lamiaceae; Agastachis herba], Morus alba L. [Moraceae; Mori folium], Bupleurum falcatum L. [Apiaceae; Bupleuri radix], Actaea spp. [Ranunculaceae; Cimicifugae rhizoma] |
| Lee 2024 | Bojungikki-tang | Atractylodes macrocephala Koidz. [Asteraceae; Atractylodis rhizoma alba], Panax ginseng C.A.Mey. [Araliaceae; Ginseng radix], Astragalus membranaceus (Fisch.) Bunge [Fabaceae; Astragali radix], Angelica gigas Nakai [Apiaceae; Angelicae gigantis radix], Citrus unshiu Marcow. [Rutaceae; Citri unshius pericarpium], Bupleurum falcatum L. [Apiaceae; Bupleuri radix], Ziziphus jujuba Mill. [Rhamnaceae; Ziziphi fructus], Glycyrrhiza uralensis Fisch. ex DC. [Fabaceae; Glycyrrhizae radix et rhizoma], Actaea spp. [Ranunculaceae; Cimicifugae rhizoma], Zingiber officinale Roscoe [Zingiberaceae; Zingiberis rhizoma] |
| Li 2021a | Modified Bojungikki-tang | Astragalus membranaceus (Fisch.) Bunge [Fabaceae; Astragali radix], Panax ginseng C.A.Mey. [Araliaceae; Ginseng radix], Atractylodes macrocephala Koidz. [Asteraceae; Atractylodis macrocephalae rhizoma], Angelica sinensis (Oliv.) Diels [Apiaceae; Angelicae sinensis radix], Citrus reticulata Blanco [Rutaceae; Citri reticulatae pericarpium], Polygala tenuifolia Willd. [Polygalaceae; Polygalae radix], Actaea heracleifolia (Kom.) J.Compton [Ranunculaceae; Cimicifugae rhizoma], Bupleurum chinense DC. [Apiaceae; Bupleuri radix], Lycium barbarum L. [Solanaceae; Lycii fructus], Cuscuta chinensis Lam. [Convolvulaceae; Cuscutae semen], Schisandra chinensis (Turcz.) Baill. [Schisandraceae; Schisandrae chinensis fructus], Poria cocos (Schw.) Wolf [Polyporaceae; Poria], Zingiber officinale Roscoe [Zingiberaceae; Zingiberis rhizoma], Ziziphus jujuba Mill. [Rhamnaceae; Ziziphi fructus], Glycyrrhiza uralensis Fisch. ex DC. [Fabaceae; Glycyrrhizae radix] |
| Li 2021b | Bojungikki-tang | NR |
| Li 2016 | Modified Bojungikki-tang added to standard radio-/chemotherapy | Astragalus membranaceus (Fisch.) Bunge [Fabaceae; Astragali radix], Hordeum vulgare L. [Poaceae; Hordei fructus germinatus], Triticum aestivum L. [Poaceae; Tritici fructus germinatus], Atractylodes macrocephala Koidz. [Asteraceae; Atractylodis macrocephalae rhizoma], Panax ginseng C.A.Mey. [Araliaceae; Ginseng radix], Citrus reticulata Blanco [Rutaceae; Citri reticulatae pericarpium], Bupleurum chinense DC. [Apiaceae; Bupleuri radix], Ligustrum lucidum W.T.Aiton [Oleaceae; Ligustri lucidi fructus], Glycyrrhiza uralensis Fisch. ex DC. [Fabaceae; Glycyrrhizae radix praeparata], Actaea heracleifolia (Kom.) J.Compton [Ranunculaceae; Cimicifugae rhizoma], Eclipta prostrata (L.) L. [Asteraceae; Ecliptae herba], Angelica sinensis (Oliv.) Diels [Apiaceae; Angelicae sinensis radix], Amomum villosum Lour. [Zingiberaceae; Amomi fructus], Ligusticum striatum DC. [Apiaceae; Chuanxiong rhizoma], Vitex trifolia L. [Lamiaceae; Viticis fructus], Paeonia lactiflora Pall. [Paeoniaceae; Paeoniae radix alba], Ophiopogon japonicus (Thunb.) Ker Gawl. [Asparagaceae; Ophiopogonis radix], Schisandra chinensis (Turcz.) Baill. [Schisandraceae; Schisandrae chinensis fructus] |
| Lu 2021 | Modified Bojungikki-tang + Kangtai ointment | Paeonia lactiflora Pall. [Paeoniaceae; Paeoniae radix alba], Atractylodes macrocephala Koidz. [Asteraceae; Atractylodis macrocephalae rhizoma], Angelica sinensis (Oliv.) Diels [Apiaceae; Angelicae sinensis radix], Panax ginseng C.A.Mey. [Araliaceae; Ginseng radix], Bupleurum spp. [Apiaceae; Bupleuri radix], Actaea spp. [Ranunculaceae; Cimicifugae rhizoma], Citrus reticulata Blanco [Rutaceae; Citri reticulatae pericarpium], Glycyrrhiza uralensis Fisch. ex DC. [Fabaceae; Glycyrrhizae radix praeparata], Astragalus membranaceus (Fisch.) Bunge [Fabaceae; Astragali radix] |
| Minagawa 2019 | Bojungikki-tang | Astragalus membranaceus (Fisch.) Bunge [Fabaceae; Astragali radix], Atractylodes japonica Koidz. [Asteraceae; Atractylodis rhizoma], Panax ginseng C.A.Mey. [Araliaceae; Ginseng radix], Angelica acutiloba (Siebold & Zucc.) Kitag. [Apiaceae; Angelicae acutilobae radix], Bupleurum falcatum L. [Apiaceae; Bupleuri radix], Ziziphus jujuba Mill. [Rhamnaceae; Ziziphi fructus], Citrus unshiu Marcow. [Rutaceae; Citri unshius pericarpium], Glycyrrhiza uralensis Fisch. ex DC. [Fabaceae; Glycyrrhizae radix], Actaea japonica Thunb. [Ranunculaceae; Cimicifugae rhizoma], Zingiber officinale Roscoe [Zingiberaceae; Zingiberis rhizoma] |
| Ni 2021 | Bojungikki-tang | Astragalus membranaceus (Fisch.) Bunge [Fabaceae; Astragali radix], Poria cocos (Schw.) Wolf [Polyporaceae; Poria], Codonopsis pilosula (Franch.) Nannf. [Campanulaceae; Codonopsis radix], Atractylodes macrocephala Koidz. [Asteraceae; Atractylodis macrocephalae rhizoma], Actaea heracleifolia (Kom.) J.Compton [Ranunculaceae; Cimicifugae rhizoma], Bupleurum chinense DC. [Apiaceae; Bupleuri radix], Citrus reticulata Blanco [Rutaceae; Citri reticulatae pericarpium], Angelica sinensis (Oliv.) Diels [Apiaceae; Angelicae sinensis radix], Acorus tatarinowii Schott [Acoraceae; Acori tatarinowii rhizoma], Salvia miltiorrhiza Bunge [Lamiaceae; Salviae miltiorrhizae radix et rhizoma], Epimedium brevicornu Maxim. [Berberidaceae; Epimedii herba], Rehmannia glutinosa (Gaertn.) DC. [Orobanchaceae; Rehmanniae radix praeparata] |
| Oh 2023 | Bojungikki-tang + glucose-lowering drugs | Astragalus membranaceus (Fisch.) Bunge [Fabaceae; Astragali radix], Atractylodes macrocephala Koidz. [Asteraceae; Atractylodis macrocephalae rhizoma], Gardenia jasminoides J.Ellis [Rubiaceae; Gardeniae fructus], Angelica gigas Nakai [Apiaceae; Angelicae gigantis radix], Glycyrrhiza uralensis Fisch. ex DC. [Fabaceae; Glycyrrhizae radix], Citrus reticulata Blanco [Rutaceae; Citri reticulatae pericarpium], Liriope platyphylla F.T.Wang & T.Tang [Asparagaceae; Liriopes radix], Magnolia biondii Pamp. [Magnoliaceae; Magnoliae flos], Panax ginseng C.A.Mey. [Araliaceae; Ginseng radix], Actaea heracleifolia (Kom.) J.Compton [Ranunculaceae; Cimicifugae rhizoma], Asarum sieboldii Miq. [Aristolochiaceae; Asiasari radix], Bupleurum falcatum L. [Apiaceae; Bupleuri radix], Zingiber officinale Roscoe [Zingiberaceae; Zingiberis rhizoma], Ziziphus jujuba Mill. [Rhamnaceae; Ziziphi fructus] |
| Okabe 2019 | Bojungikki-tang + S-1 adjuvant chemotherapy | Astragalus membranaceus (Fisch.) Bunge [Fabaceae; Astragali radix], Atractylodes macrocephala Koidz. [Asteraceae; Atractylodis macrocephalae rhizoma], Panax ginseng C.A.Mey. [Araliaceae; Ginseng radix], Angelica acutiloba (Siebold & Zucc.) Kitag. [Apiaceae; Angelicae acutilobae radix], Bupleurum chinense DC. [Apiaceae; Bupleuri radix], Ziziphus jujuba Mill. [Rhamnaceae; Ziziphi fructus], Citrus unshiu Marcow. [Rutaceae; Citri unshius pericarpium], Glycyrrhiza uralensis Fisch. ex DC. [Fabaceae; Glycyrrhizae radix], Actaea heracleifolia (Kom.) J.Compton [Ranunculaceae; Cimicifugae rhizoma], Zingiber officinale Roscoe [Zingiberaceae; Zingiberis rhizoma] |
| Okugawa 2024 | Bojungikki-tang | Astragalus membranaceus (Fisch.) Bunge [Fabaceae; Astragali radix], Atractylodes lancea (Thunb.) DC. [Asteraceae; Atractylodis lanceae rhizoma], Panax ginseng C.A.Mey. [Araliaceae; Ginseng radix], Angelica acutiloba (Siebold & Zucc.) Kitag. [Apiaceae; Angelicae acutilobae radix], Bupleurum chinense DC. [Apiaceae; Bupleuri radix], Ziziphus jujuba Mill. [Rhamnaceae; Ziziphi fructus], Citrus unshiu Marcow. [Rutaceae; Citri unshius pericarpium], Glycyrrhiza uralensis Fisch. ex DC. [Fabaceae; Glycyrrhizae radix], Actaea heracleifolia (Kom.) J.Compton [Ranunculaceae; Cimicifugae rhizoma], Zingiber officinale Roscoe [Zingiberaceae; Zingiberis rhizoma] |
| Park 2025 | Modified Bojungikki-tang | Astragalus membranaceus (Fisch.) Bunge [Fabaceae; Astragali radix], Angelica gigas Nakai [Apiaceae; Angelicae gigantis radix], Cervus elaphus L. [Cervidae; Cervi cornu colla], Panax ginseng C.A.Mey. [Araliaceae; Ginseng radix], Atractylodes macrocephala Koidz. [Asteraceae; Atractylodis macrocephalae rhizoma], Glycyrrhiza uralensis Fisch. ex DC. [Fabaceae; Glycyrrhizae radix], Equus asinus L. [Equidae; Asini corii colla], Rehmannia glutinosa (Gaertn.) DC. [Orobanchaceae; Rehmanniae radix], Actaea heracleifolia (Kom.) J.Compton [Ranunculaceae; Cimicifugae rhizoma], Bupleurum falcatum L. [Apiaceae; Bupleuri radix], Citrus unshiu Marcow. [Rutaceae; Citri unshius pericarpium], Poria cocos (Schw.) Wolf [Polyporaceae; Poria], Alisma orientale (Sam.) Juz. [Alismataceae; Alismatis rhizoma], Zingiber officinale Roscoe [Zingiberaceae; Zingiberis rhizoma] |
| Qian 2024 | Bojungikki-tang | Astragalus membranaceus (Fisch.) Bunge [Fabaceae; Astragali radix], Codonopsis pilosula (Franch.) Nannf. [Campanulaceae; Codonopsis radix], Atractylodes macrocephala Koidz. [Asteraceae; Atractylodis macrocephalae rhizoma], Angelica sinensis (Oliv.) Diels [Apiaceae; Angelicae sinensis radix], Actaea cimicifuga L. [Ranunculaceae; Cimicifugae rhizoma], Bupleurum chinense DC. [Apiaceae; Bupleuri radix], Citrus reticulata Blanco [Rutaceae; Citri reticulatae pericarpium], Glycyrrhiza uralensis Fisch. ex DC. [Fabaceae; Glycyrrhizae radix] |
| Qiu 2024 | Modified Bojungikki-tang | Astragalus membranaceus (Fisch.) Bunge [Fabaceae; Astragali radix], Angelica sinensis (Oliv.) Diels [Apiaceae; Angelicae sinensis radix], Atractylodes macrocephala Koidz. [Asteraceae; Atractylodis macrocephalae rhizoma], Codonopsis pilosula (Franch.) Nannf. [Campanulaceae; Codonopsis radix], Citrus reticulata Blanco [Rutaceae; Citri reticulatae pericarpium], Glycyrrhiza uralensis Fisch. ex DC. [Fabaceae; Glycyrrhizae radix], Panax ginseng C.A.Mey. [Araliaceae; Ginseng radix], Actaea cimicifuga L. [Ranunculaceae; Cimicifugae rhizoma], Bupleurum chinense DC. [Apiaceae; Bupleuri radix], Dioscorea polystachya Turcz. [Dioscoreaceae; Dioscoreae rhizoma], Epimedium brevicornu Maxim. [Berberidaceae; Epimedii herba], Coix lacryma-jobi L. [Poaceae; Coicis semen], Lablab purpureus (L.) Sweet [Fabaceae; Lablab semen album], Euryale ferox Salisb. [Nymphaeaceae; Euryales semen], Bombyx batryticatus [Bombycidae; Bombyx batryticatus], Scolopendra subspinipes Leach [Scolopendridae; Scolopendra] |
| Seo 2019 | Bojungikki-tang | Astragalus membranaceus (Fisch.) Bunge [Fabaceae; Astragali radix], Panax ginseng C.A.Mey. [Araliaceae; Ginseng radix], Atractylodes macrocephala Koidz. [Asteraceae; Atractylodis rhizoma alba], Glycyrrhiza uralensis Fisch. ex DC. [Fabaceae; Glycyrrhizae radix], Angelica gigas Nakai [Apiaceae; Angelicae gigantis radix], Citrus reticulata Blanco [Rutaceae; Aurantii nobilis pericarpium], Actaea spp. [Ranunculaceae; Cimicifugae rhizoma], Bupleurum falcatum L. [Apiaceae; Bupleuri radix] |
| Takeuchi 2017 | Bojungikki-tang | NR |
| Teng 2016 | Modified Bojungikki-tang + norethindrone | Astragalus membranaceus (Fisch.) Bunge [Fabaceae; Astragali radix], Atractylodes macrocephala Koidz. [Asteraceae; Atractylodis macrocephalae rhizoma], Citrus reticulata Blanco [Rutaceae; Citri reticulatae pericarpium], Actaea cimicifuga L. [Ranunculaceae; Cimicifugae rhizoma], Bupleurum chinense DC. [Apiaceae; Bupleuri radix], Codonopsis pilosula (Franch.) Nannf. [Campanulaceae; Codonopsis radix], Glycyrrhiza uralensis Fisch. ex DC. [Fabaceae; Glycyrrhizae radix praeparata], Pueraria lobata (Willd.) Ohwi [Fabaceae; Puerariae lobatae radix], Dipsacus asper Wall. ex C.B.Clarke [Caprifoliaceae; Dipsaci radix] |
| Terai 2020 | Bojungikki-tang | NR |
| Tokumasu 2025 | Bojungikki-tang | Panax ginseng C.A.Mey. [Araliaceae; Ginseng radix], Ziziphus jujuba Mill. [Rhamnaceae; Ziziphi fructus], Atractylodes spp. [Asteraceae; Atractylodis rhizoma], Bupleurum spp. [Apiaceae; Bupleuri radix], Astragalus membranaceus (Fisch.) Bunge [Fabaceae; Astragali radix], Glycyrrhiza uralensis Fisch. ex DC. [Fabaceae; Glycyrrhizae radix], Angelica gigas Nakai [Apiaceae; Angelicae gigantis radix], Zingiber officinale Roscoe [Zingiberaceae; Zingiberis rhizoma], Citrus reticulata Blanco [Rutaceae; Aurantii nobilis pericarpium], Actaea spp. [Ranunculaceae; Cimicifugae rhizoma], Atractylodes lancea (Thunb.) DC. [Asteraceae; Atractylodis lanceae rhizoma] |
| Wang 2016 | Modified Bojungikki-tang | Codonopsis pilosula (Franch.) Nannf. [Campanulaceae; Codonopsis radix], Astragalus membranaceus (Fisch.) Bunge [Fabaceae; Astragali radix], Atractylodes macrocephala Koidz. [Asteraceae; Atractylodis macrocephalae rhizoma], Angelica sinensis (Oliv.) Diels [Apiaceae; Angelicae sinensis radix], Citrus reticulata Blanco [Rutaceae; Citri reticulatae pericarpium], Bupleurum chinense DC. [Apiaceae; Bupleuri radix], Actaea spp. [Ranunculaceae; Cimicifugae rhizoma], Glycyrrhiza uralensis Fisch. ex DC. [Fabaceae; Glycyrrhizae radix praeparata] |
| Wang 2021 | Bojungikki-tang + THP bladder perfusion | Astragalus membranaceus (Fisch.) Bunge [Fabaceae; Astragali radix], Panax ginseng C.A.Mey. [Araliaceae; Ginseng radix], Angelica gigas Nakai [Apiaceae; Angelicae gigantis radix], Glycyrrhiza uralensis Fisch. ex DC. [Fabaceae; Glycyrrhizae radix], Atractylodes macrocephala Koidz. [Asteraceae; Atractylodis macrocephalae rhizoma], Actaea cimicifuga L. [Ranunculaceae; Cimicifugae rhizoma], Citrus reticulata Blanco [Rutaceae; Citri reticulatae pericarpium], Bupleurum chinense DC. [Apiaceae; Bupleuri radix] |
| Watanabe 2022 | Bojungikki-tang | Astragalus membranaceus (Fisch.) Bunge [Fabaceae; Astragali radix], Atractylodes lancea (Thunb.) DC. [Asteraceae; Atractylodis lanceae rhizoma], Panax ginseng C.A.Mey. [Araliaceae; Ginseng radix], Angelica acutiloba (Siebold & Zucc.) Kitag. [Apiaceae; Angelicae acutilobae radix], Bupleurum falcatum L. [Apiaceae; Bupleuri radix], Ziziphus jujuba Mill. [Rhamnaceae; Ziziphi fructus], Citrus unshiu Marcow. [Rutaceae; Citri unshius pericarpium], Glycyrrhiza uralensis Fisch. ex DC. [Fabaceae; Glycyrrhizae radix], Actaea simplex (DC.) Wormsk. ex Prantl [Ranunculaceae; Cimicifugae rhizoma], Zingiber officinale Roscoe [Zingiberaceae; Zingiberis rhizoma] |
| Wu 2017 | Bojungikki-tang | Astragalus membranaceus (Fisch.) Bunge [Fabaceae; Astragali radix], Codonopsis pilosula (Franch.) Nannf. [Campanulaceae; Codonopsis radix], Atractylodes macrocephala Koidz. [Asteraceae; Atractylodis macrocephalae rhizoma], Angelica sinensis (Oliv.) Diels [Apiaceae; Angelicae sinensis radix], Citrus reticulata Blanco [Rutaceae; Citri reticulatae pericarpium], Bupleurum chinense DC. [Apiaceae; Bupleuri radix], Actaea cimicifuga L. [Ranunculaceae; Cimicifugae rhizoma], Glycyrrhiza uralensis Fisch. ex DC. [Fabaceae; Glycyrrhizae radix praeparata] |
| Xu 2023 | Bojungikki-tang + conventional Western medicine | Astragalus membranaceus (Fisch.) Bunge [Fabaceae; Astragali radix], Panax ginseng C.A.Mey. [Araliaceae; Ginseng radix], Glycyrrhiza uralensis Fisch. ex DC. [Fabaceae; Glycyrrhizae radix], Atractylodes macrocephala Koidz. [Asteraceae; Atractylodis macrocephalae rhizoma], Angelica sinensis (Oliv.) Diels [Apiaceae; Angelicae sinensis radix], Citrus reticulata Blanco [Rutaceae; Citri reticulatae pericarpium], Actaea spp. [Ranunculaceae; Cimicifugae rhizoma], Bupleurum chinense DC. [Apiaceae; Bupleuri radix] |
| Yang 2016 | Mosapride + Bojungikki-tang | NR |
| Zhang 2015 | Modified Bojungikki-tang + topical powder | Astragalus membranaceus (Fisch.) Bunge [Fabaceae; Astragali radix], Codonopsis pilosula (Franch.) Nannf. [Campanulaceae; Codonopsis radix], Atractylodes macrocephala Koidz. [Asteraceae; Atractylodis macrocephalae rhizoma], Glycyrrhiza uralensis Fisch. ex DC. [Fabaceae; Glycyrrhizae radix], Angelica sinensis (Oliv.) Diels [Apiaceae; Angelicae sinensis radix], Citrus reticulata Blanco [Rutaceae; Citri reticulatae pericarpium], Poria cocos (Schw.) Wolf [Polyporaceae; Poria], Bupleurum chinense DC. [Apiaceae; Bupleuri radix], Actaea cimicifuga L. [Ranunculaceae; Cimicifugae rhizoma], Sanguisorba officinalis L. [Rosaceae; Sanguisorbae radix], Rubia cordifolia L. [Rubiaceae; Rubiae radix et rhizoma] |
| Zhou 2022 | Modified Bojungikki-tang | Astragalus membranaceus (Fisch.) Bunge [Fabaceae; Astragali radix], Atractylodes macrocephala Koidz. [Asteraceae; Atractylodis macrocephalae rhizoma], Citrus reticulata Blanco [Rutaceae; Citri reticulatae pericarpium], Actaea cimicifuga L. [Ranunculaceae; Cimicifugae rhizoma], Bupleurum chinense DC. [Apiaceae; Bupleuri radix], Panax ginseng C.A.Mey. [Araliaceae; Ginseng radix], Glycyrrhiza uralensis Fisch. ex DC. [Fabaceae; Glycyrrhizae radix], Angelica sinensis (Oliv.) Diels [Apiaceae; Angelicae sinensis radix], Alpinia oxyphylla Miq. [Zingiberaceae; Alpiniae oxyphyllae fructus], Mantidis ootheca [Mantidae; Mantidis ootheca], Eucommia ulmoides Oliv. [Eucommiaceae; Eucommiae cortex], Lindera aggregata (Sims) Kosterm. [Lauraceae; Linderae radix], Rosa laevigata Michx. [Rosaceae; Rosae laevigatae fructus] |

Scientific names, author citations, family names, and pharmacopeial drug names were standardized where identifiable using the information reported in the original studies. Materials not reported or not identifiable at the species level were marked as NR or retained at the genus level. Animal-derived and non-botanical materials were retained when included in modified formulations. NR, not reported.
